# Inflammatory mediators of mRNA vaccine-induced adverse reactions in mice

**DOI:** 10.1016/j.ymthe.2026.01.022

**Published:** 2026-01-20

**Authors:** Koyo Honda, Tatsuya Karaki, Yuta Kunishima, Yoshino Kawaguchi, Naoki Takemura, Takashi Matsuzaki, So-ichiro Fukada, Tatsuya Saitoh, Toshiro Hirai, Yasuo Yoshioka

**Affiliations:** 1 Laboratory of Nano-design for Innovative Drug Development, Graduate School of Pharmaceutical Sciences, The University of Osaka, 1-6 Yamadaoka, Suita, Osaka 565-0871, Japan; 2 Vaccine Creation Group, BIKEN Innovative Vaccine Research Alliance Laboratories, Research Institute for Microbial Diseases, The University of Osaka, 3-1 Yamadaoka, Suita, Osaka 565-0871, Japan; 3 The Research Foundation for Microbial Diseases of Osaka University, 3-1 Yamadaoka, Suita, Osaka 565-0871, Japan; 4 Laboratory of Bioresponse Regulation, Graduate School of Pharmaceutical Sciences, The University of Osaka, 1-6 Yamadaoka, Suita, Osaka 565-0871, Japan; 5 Department of DDS Pharmaceutical Development, Graduate School of Medicine, The University of Osaka, 2-2 Yamadaoka, Suita, Osaka 565-0871, Japan; 6 Center for Advanced Modalities and DDS, The University of Osaka, 3-1 Yamadaoka, Suita, Osaka 565-0871, Japan; 7 Laboratory of Stem Cell Regeneration and Adaptation, Graduate School of Pharmaceutical Sciences, The University of Osaka, 1-6 Yamadaoka, Suita, Osaka 565-0871, Japan; 8 Center for Infectious Disease Education and Research, The University of Osaka, 3-1 Yamadaoka, Suita, Osaka 565-0871, Japan; 9 Global Center for Medical Engineering and Informatics, The University of Osaka, 3-1 Yamadaoka, Suita, Osaka 565-0871, Japan; 10 Vaccine Creation Group, BIKEN Innovative Vaccine Research Alliance Laboratories, Institute for Open and Transdisciplinary Research Initiatives, The University of Osaka, 3-1 Yamadaoka, Suita, Osaka 565-0871, Japan

**Keywords:** adverse reactions, cytokines, fever, IL-6, lipid nanoparticle, mRNA vaccine

## Abstract

mRNA vaccines are promising vaccine modalities against infectious diseases. However, these vaccines frequently cause adverse reactions, such as fever and fatigue, which are exacerbated after a booster dose, leading to vaccine hesitancy. Here, we elucidated the mechanisms underlying adverse reactions in mice after prime and boost vaccinations with mRNA vaccines. The mRNA vaccine induced systemic adverse reactions, such as fever, and local adverse reactions, such as enhanced vascular permeability, at the vaccination site in mice. Lipid nanoparticles (LNPs) used for mRNA encapsulation mainly contributed to these adverse reactions. We identified IL-1, IL-6, TNF-α, and type 1 IFN as key cytokines and COX-2 and PGE2 as inflammatory mediators responsible for systemic adverse reactions. TNF-α levels were enhanced after the boost vaccination by IFN-γ secreted from prime-vaccination-induced T cells, contributing to systemic adverse reactions. In addition, local adverse reactions at the vaccination site were caused by a mechanism different from that of systemic adverse reactions. We also found that the inhibition of IL-6 effectively reduced the adverse reactions while maintaining the vaccine effects. These data provide basic information for understanding adverse reactions in humans and may be useful for developing mRNA vaccines with fewer adverse reactions.

## Graphical abstract

**Figure:**
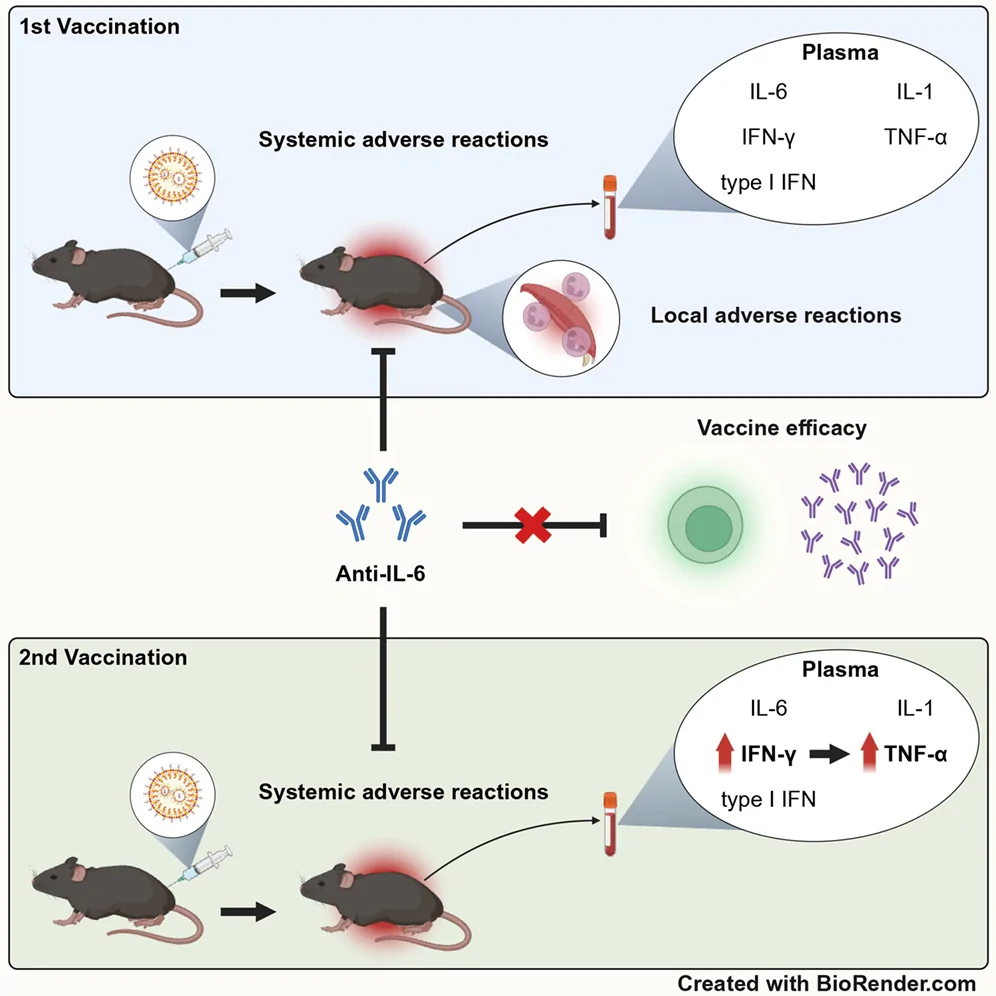

## Teaser

Yoshioka and colleagues elucidate the mechanisms underlying adverse reactions induced by prime and boost immunizations with an mRNA vaccine in mice. These findings provide insights into the adverse reactions associated with mRNA vaccines in humans and may inform the development of mRNA vaccine formulations with reduced adverse effects.

## Introduction

In recent years, outbreaks of infectious diseases have seriously impacted the world. The emerging infectious diseases require urgent development of reliable and safe vaccines. Compared with conventional vaccines, such as live attenuated and inactivated vaccines, messenger RNA (mRNA) vaccines have emerged as a promising platform.^1^^,^^2^^,^^3^^,^^4^ mRNA vaccines can be developed rapidly by obtaining gene sequences for pathogen-derived proteins, enabling a rapid response to emergencies such as pandemics.^1^^,^^2^^,^^3^^,^^4^ mRNA vaccines developed by Pfizer/BioNTech and Moderna played a critical role in controlling the COVID-19 pandemic caused by SARS-CoV-2 in 2019.^5^ In addition, mRNA vaccines have been used for a variety of infectious diseases; these include an mRNA vaccine approved for the respiratory syncytial virus and another for influenza virus, which is in development.^6^

mRNA vaccines induce robust antigen-specific antibody production and T cell responses that prevent infection and severe diseases.^1^ However, the mRNA vaccines often cause systemic adverse reactions, including fever and fatigue, and localized adverse reactions, such as pain and swelling, at the vaccination site.^7^^,^^8^^,^^9^^,^^10^^,^^11^^,^^12^ The frequency and severity of these adverse reactions increase with repeated vaccinations, although multiple vaccinations enhance antigen-specific immune responses.^10^^,^^13^^,^^14^ The occurrence of adverse reactions has led to vaccine hesitancy and fear, and this aversion can prevent the application of mRNA vaccines against future human threats.^15^^,^^16^ Therefore, addressing these adverse reactions is crucial for improving public acceptance.

In humans, blood levels of inflammatory cytokines, such as interleukin (IL)-6 and tumor necrosis factor (TNF)-α, are increased after mRNA vaccine administration.^14^^,^^17^^,^^18^ Furthermore, blood levels of cytokines such as IL-6 correlate with the severity of adverse reactions following mRNA vaccination, suggesting the importance of inflammatory cytokines in inducing adverse reactions.^14^ Antigen-specific immune responses induced by mRNA vaccines are also part of the mechanism underlying the increased frequency and severity of adverse reactions after boost vaccination in humans. For example, the rate of increase in the abundance of type 1 T helper (Th1) cells in the blood after boost vaccination correlates with a high frequency of adverse reactions,^19^ and the concentration of interferon (IFN)-γ and the titer of neutralizing antibodies in the blood strongly correlate with the frequency and severity of adverse reactions.^14^ The inflammatory properties of mRNA vaccines have also been studied in mice; levels of inflammatory cytokines such as IL-6 are increased in the blood of mice after administration of mRNA vaccines.^20^^,^^21^ However, previous reports on humans and mice have been limited to studies reporting the correlation between the frequency and severity of adverse reactions, the cytokine levels, and the specific cell number, and only a few studies have directly examined the factors that cause adverse reactions.^14^^,^^22^^,^^23^ In addition, most studies in mice have focused on inflammatory reactions after the prime vaccination, leaving the reason for the increased rate of adverse reactions after boost vaccination unexplored. Therefore, understanding the precise mechanisms of adverse reactions after prime and boost vaccinations is critical for designing mRNA vaccines with fewer adverse reactions.

Here, we attempted to elucidate the mechanisms underlying adverse reactions after mRNA vaccination in mice. We identified some cytokines and specific cells that play crucial roles in the induction of adverse reactions after prime and boost vaccinations. Our findings provide a basis for developing next-generation mRNA vaccines with reduced adverse reactions and overcoming vaccine hesitancy.

## Results

### Adverse reactions following mRNA vaccination in mice

**Figure 1:**
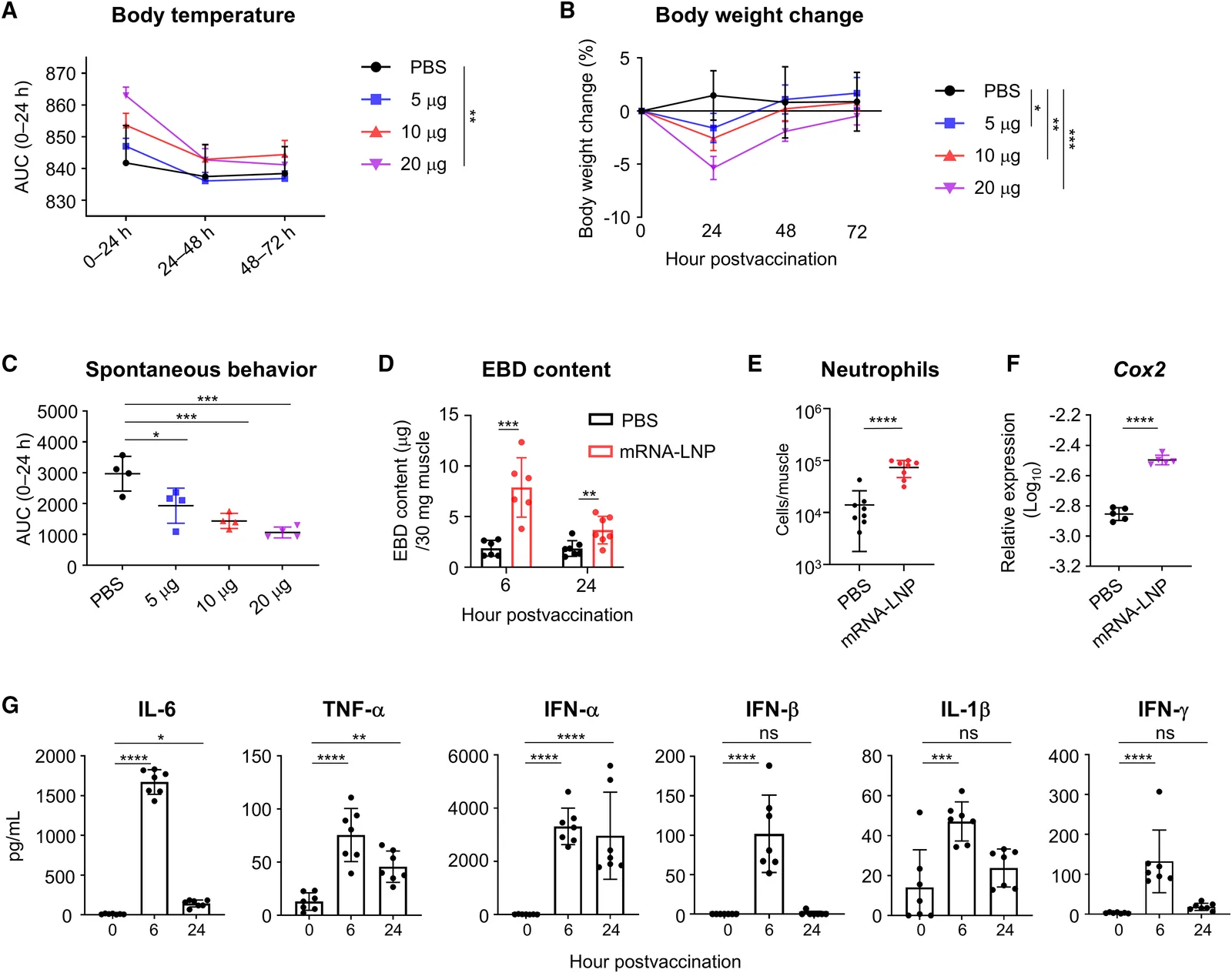
Adverse reactions induced by mRNA vaccine in mice (A–C) Mice were vaccinated intramuscularly with PBS or 5, 10, or 20 μg mRNA-LNPs, and the following parameters were measured: (A) area under the curve (AUC) for body temperature change every 24 h over 72 h, (B) changes in body weight every 24 h over 72 h, and (C) AUC for behavioral changes over 24 h. (D and E) Mice were administered 10 μg mRNA-LNPs in the anterior tibialis muscle. (D) The content of Evans blue dye (EBD) in muscle was measured for 6 and 24 h. (E) Number of neutrophils in the muscle after 6 h was assessed using flow cytometry. (F) Relative expression levels of *Cox2* mRNA, normalized to *Gapdh*, in the brain were quantified 6 h after administering PBS or 20 μg mRNA-LNPs using real-time PCR. (G) Mice were vaccinated intramuscularly with 20 μg mRNA-LNPs. Plasma levels of cytokines were measured at 6 and 24 h after vaccination. (A–C) *n* = 4, (D) *n* = 6–7, (E) *n* = 8, (F) *n* = 5, and (G) *n* = 7. (D, E, and G) Data represent pooled results of two independent experiments. (A–G) Data are expressed as means ± SD; ∗*p* < 0.05, ∗∗*p* < 0.01, ∗∗∗*p* < 0.001, and ∗∗∗∗*p* < 0.0001 by (A–C and G) Dunnett’s multiple comparison test vs. PBS at (A) 0–24 h, (B) 24 h, (G) 0 h or by (D–F) Student’s *t* test. ns, not significant.

Currently approved mRNA vaccine formulations encapsulate antigen-encoding mRNA in lipid nanoparticles (LNPs). LNPs are composed of four main components: ionizable lipid, phospholipid, cholesterol, and polyethylene glycol (PEG)-conjugated lipid. We used an mRNA encoding the full-length spike protein of SARS-CoV-2. All uridines were replaced with N1-methylpseudouridine, and the 5′ end of the mRNA was modified with Cap-1. We prepared LNP-encapsulated mRNA (mRNA-LNP) by mixing SM-102/DSPC/cholesterol/PEG_2000_-DMG (ionizable lipid/distearoylphosphatidylcholine/cholesterol/PEG-conjugated lipid) at a ratio of 50/10/38.5/1.5 with the mRNA. To evaluate whether adverse reactions such as fever, reported in humans following mRNA vaccination, would also occur in mice, we administered them intramuscular doses of 5, 10, and 20 μg mRNA-LNPs and monitored their body temperature, body weight changes, and spontaneous behavioral shifts. Over a 72-h period, the mRNA-LNP-treated groups, especially that administered 20 μg mRNA-LNPs, showed fever by 24 h after vaccination (Figures 1A, S1A, and S1B). Peak fever was observed at 5–7 h during the 24-h period (Figures S1A and S1B). The mRNA-LNP groups also showed significant body weight loss 24 h after vaccination, whereas no change was observed in the phosphate-buffered saline (PBS) group (Figure 1B). Body weight loss was maximum at 24 h and recovered at 72 h (Figure 1B). In addition, food intake within 24 h of mRNA-LNP vaccination decreased in a dose-dependent manner (Figure S1C) and the rate of food intake was strongly and inversely related to body weight loss (Figure S1D). In addition, a significant reduction in spontaneous behavior was observed in the mRNA-LNP group compared with the PBS group (Figure 1C).

In humans, pain and swelling at the vaccination site occur after mRNA vaccination.^10^^,^^13^ To investigate local inflammation in mice, we used Evans blue dye to assess the increase in vascular permeability of muscles at the vaccination site. Evans blue leakage from the muscle in the mRNA-LNP group increased significantly at 6 and 24 h compared with that in the PBS group, indicating higher vascular permeability (Figure 1D). At 6 h, when vascular permeability peaked, a marked infiltration of Ly6G^+^ neutrophils in the muscle was noted compared with that in the PBS group (Figures 1E and S2).

During fever induced by inflammation, inflammatory cytokines induce cyclooxygenase-2 (COX-2) expression in the brain.^24^ COX-2 is involved in the production of prostaglandins, which cause fever.^24^ We confirmed the significantly increased expression of COX-2 mRNA in mouse brain in the mRNA-LNP group compared with the PBS group (Figure 1F). Therefore, we focused on cytokines in the blood and measured their levels at 6 and 24 h after vaccination. Blood levels of inflammatory cytokines, IL-6, TNF-α, IFN-α, IFN-β, IL-1β, and IFN-γ, were significantly increased in the mRNA-LNP group compared with the pretreatment levels (Figures 1G and S3A). IL-1α levels were below the detection limit in the majority of blood samples (Figure S3A). Moreover, real-time polymerase chain reaction (PCR) analysis revealed remarkable mRNA expression of cytokines, such as *Il6*, *Tnfa*, *Ifna*, *Ifnb*, *Il1a*, *Il1b*, and *Ifng*, in the muscle and draining lymph nodes (Figure S3B). We also examined the mRNA expression of some cytokines in the liver and spleen (Figure S3B). Although the experiments described above were conducted using female mice, we compared the body temperature and blood levels of inflammatory cytokines after vaccination between female and male mice. In contrast to female mice, male mice did not show a significant elevation in body temperature following vaccination (Figures S4A–S4C). In addition, the levels of some inflammatory cytokines, such as IL-6, IFN-α, and IFN-γ, in male mice were significantly lower than those in female mice (Figure S4D). Collectively, these results suggest that several key features of systemic and local adverse reactions observed in humans were recapitulated in mice following mRNA vaccination, along with systemic induction of inflammatory cytokines.

### Contribution of LNPs to induction of adverse reactions

**Figure 2:**
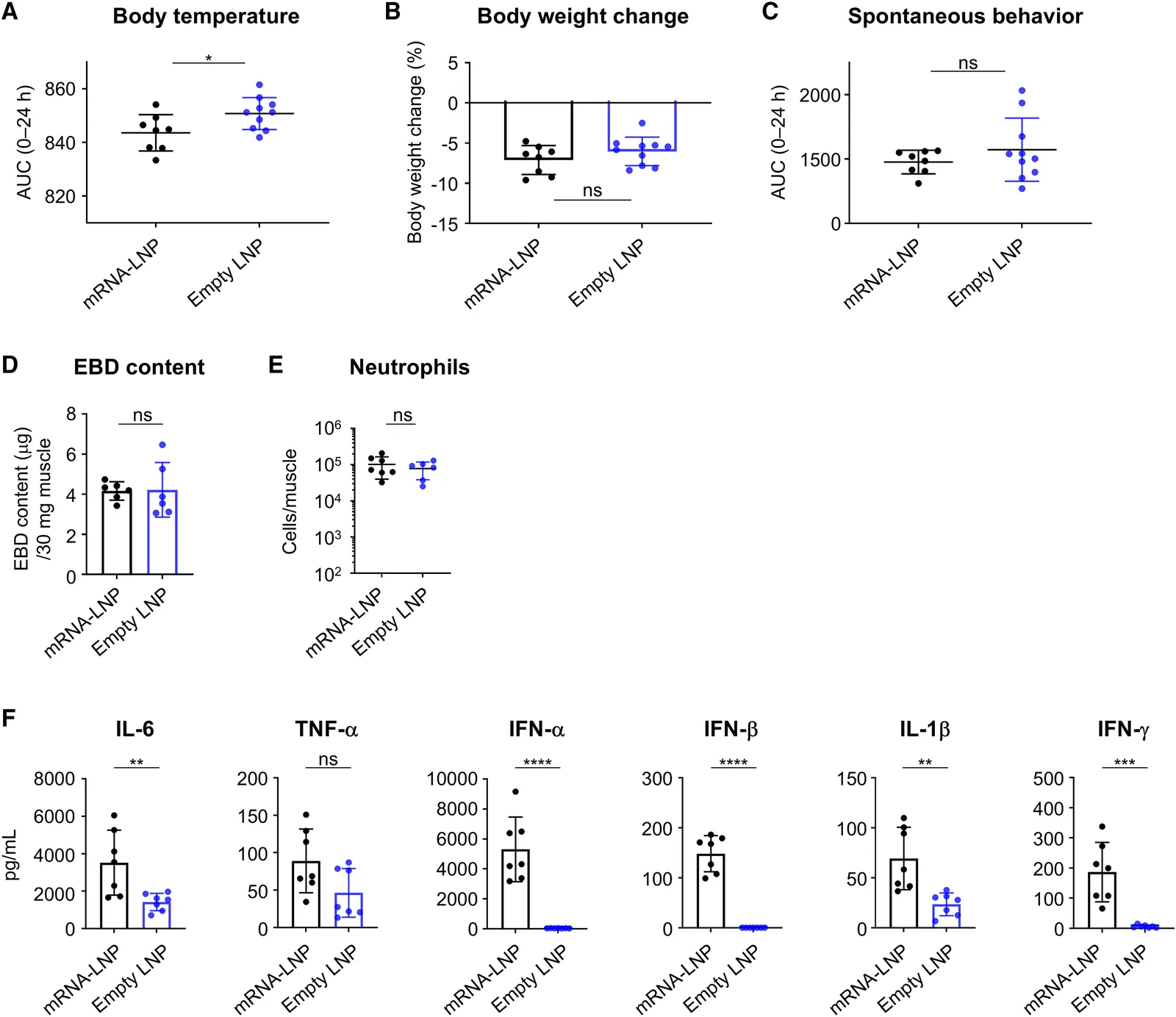
Contribution of LNPs to induction of adverse reactions (A–C) Mice were vaccinated intramuscularly with 20 μg mRNA-LNPs or empty LNPs, and the following parameters were measured over 24 h: (A) area under the curve (AUC) for body temperature change, (B) changes in body weight, and (C) AUC for behavioral changes. (D and E) Mice were administered 10 μg mRNA-LNPs or empty LNPs in the anterior tibialis muscle. (D) Evans blue dye (EBD) content in muscle was measured at 6 h. (E) Number of neutrophils in the muscle after 6 h was assessed using flow cytometry. (F) Mice were intramuscularly injected with 20 μg mRNA-LNPs or empty LNPs. Plasma levels of cytokines were measured at 6 h after mRNA-LNP vaccination. (A–C) *n* = 8–10, (D) *n* = 6, (E) *n* = 6–7, and (F) *n* = 7. (A–C, E, and F) Data represent pooled results of two independent experiments. (A–F) Data are expressed as means ± SD; ∗*p* < 0.05, ∗∗*p* < 0.01, ∗∗∗*p* < 0.001, and ∗∗∗∗*p* < 0.0001 by Student’s *t* test. ns, not significant.

To determine whether the adverse reactions induced by mRNA-LNPs were caused by LNPs alone, we compared the adverse reactions induced by mRNA-LNPs and empty LNPs without mRNA. Empty LNPs caused fever (Figures 2A and S5) and body weight loss (Figure 2B) and decreased spontaneous behavior (Figure 2C), vascular permeability (Figure 2D), and neutrophil infiltration at the vaccination site (Figure 2E), comparable to mRNA-LNP. We also compared the levels of cytokines induced by mRNA-LNPs and empty LNPs. The levels of IL-6 and IL-1β in the empty LNP group were half of those in the mRNA-LNP group, whereas type 1 IFN and IFN-γ were not induced at all in the former, suggesting that mRNA plays a role in cytokine production (Figure 2F). These results indicated that the adverse reactions induced by mRNA-LNPs were mainly due to LNPs; however, mRNA may also be involved.

### Key cytokines required for induction of systemic adverse reactions

**Figure 3:**
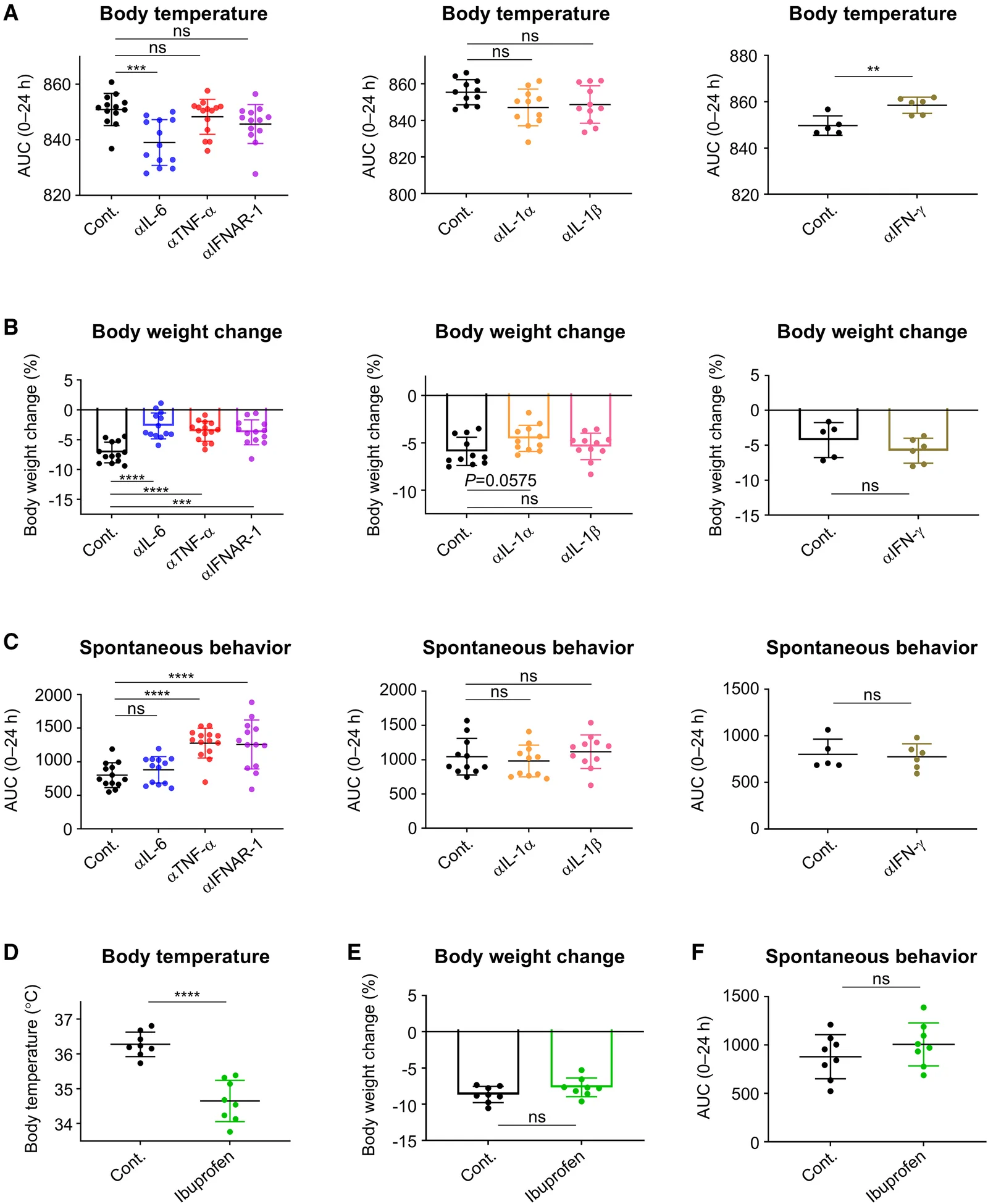
Cytokines affecting systemic adverse reactions after the prime vaccination Mice were administered a neutralizing antibody 24 h before intramuscular injection of 20 μg mRNA-LNPs. (A–C) After vaccination, the following parameters were measured over 24 h: (A) area under the curve (AUC) for body temperature change, (B) changes in body weight, and (C) AUC of behavioral changes. (D–F) Mice were intramuscularly injected with 20 μg mRNA-LNPs along with ibuprofen or 10% DMSO as a control administered 30 min before and 6 h after vaccination. After vaccination, the following parameters were measured: (D) body temperature at 7 h, (E) changes in body weight over 24 h, and (F) AUC for behavioral changes over 24 h. (A–C) *n* = 5–14 and (D–F) *n* = 8. (A–C) With the exception of IFN-γ experiments, data are pooled results from two or three independent experiments. (D–F) Data represent pooled results of two independent experiments. (A–F) Data are expressed as means ± SD; ∗∗*p* < 0.01, ∗∗∗*p* < 0.001, and ∗∗∗∗*p* < 0.0001 by Dunnett’s multiple comparison test vs. control in (A)–(C) (except for IFN-γ experiments) or by Student’s *t* test in (A)–(C) (in IFN-γ experiments) and (D)–(F). ns, not significant.

We aimed to identify the key cytokines responsible for systemic adverse reactions following mRNA vaccination. We administered neutralizing antibodies against cytokines or their receptors (IL-6, TNF-α, IFN-α/β receptor 1 [IFNAR-1], IL-1α, IL-1β, or IFN-γ) before mRNA-LNP vaccination and then measured the body temperature, body weight, and spontaneous behavior. Neutralizing antibody against IL-6 significantly reduced fever (Figures 3A and S6A). On the contrary, no significant suppression of fever was observed upon administration of neutralizing antibody against TNF-α, IFNAR-1, IL-1α, or IL-1β (Figures 3A, S6A, and S6B). Neutralizing antibodies against IL-6, TNF-α, and IFNAR-1 significantly suppressed the loss in body weight and those against IL-1α showed a decreasing trend (*p* = 0.0575); however, neutralizing antibody against IL-1β had no such effect (Figures 3B, S6A, and S6B). In addition, neutralizing antibody against IFN-γ had no effect on fever or weight loss (Figures 3A, 3B, and S6C). Furthermore, only antibodies against TNF-α and IFNAR-1 significantly restored spontaneous behavior, although the difference was minimal (Figure 3C). The stimulator of IFN genes (STING), an upstream factor for type 1 IFN production, had no effect on fever or weight loss in STING-knockout (KO) mice (Figures S7A and S7B). These findings indicated that IL-6, TNF-α, type 1 IFN, and IL-1α are key cytokines induced by mRNA-LNPs that influence systemic adverse reactions, such as fever and body weight loss.

As COX-2 was induced in the brain by mRNA-LNP vaccination (Figure 1F), we hypothesized that prostaglandin E2 (PGE2) induced by COX-2 was important for the systemic adverse reactions induced by mRNA-LNPs. To confirm this, we administered ibuprofen, a clinically used nonsteroidal anti-inflammatory drug that inhibits PGE2 production, and evaluated its effect on systemic adverse reactions. We observed a transient suppression of fever after the second administration of ibuprofen, which indicated that PGE2 contributes to the mRNA-LNP-induced fever (Figures 3D and S8). In contrast, no effects on body weight loss or spontaneous behavior were observed (Figures 3E and 3F). These data suggest that PGE2 contributes to the mRNA-LNP-induced fever.

### Effects of cytokines on local adverse reactions

**Figure 4:**
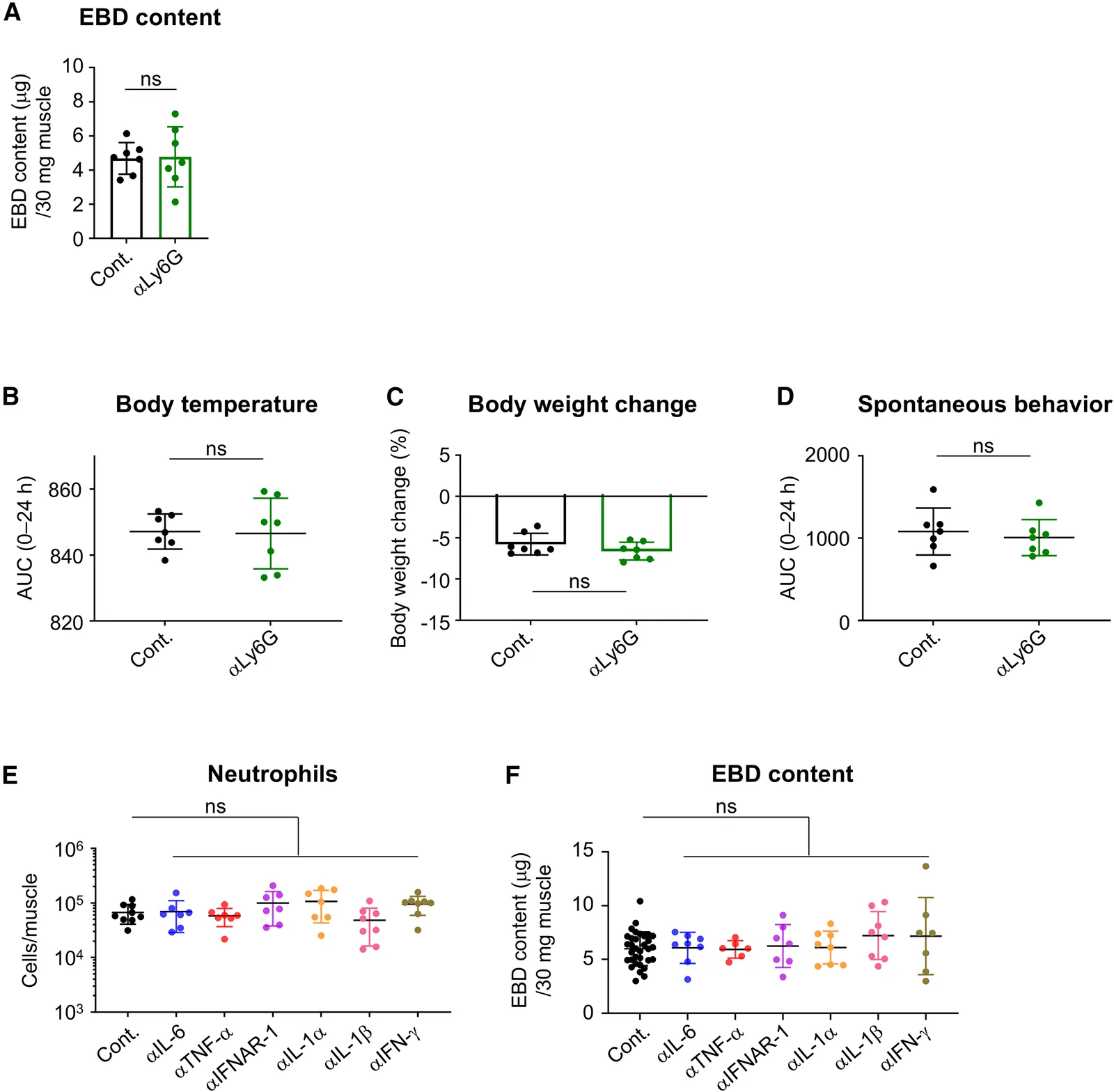
Cytokines affecting local adverse reactions (A–D) Mice were vaccinated intramuscularly with (A) 10 or (B–D) 20 μg mRNA-LNPs. Mice were administered anti-Ly6G antibody 2 days and 1 day before mRNA-LNP vaccination. (E and F) Mice were administered 10 μg mRNA-LNPs in the anterior tibialis muscle. (E) Mice were administered neutralizing antibody 24 h before mRNA-LNP vaccination. (F) Mice were intraperitoneally administered neutralizing antibody 24 h before mRNA-LNP vaccination and intramuscularly simultaneously with mRNA-LNP vaccination. (A and F) Evans blue dye (EBD) content in muscle was measured 6 h after mRNA-LNP vaccination. (B–D) After vaccination, (B) area under the curve (AUC) for body temperature change over 24 h, (C) body weight changes over 24 h, and (D) AUC for behavior change over 24 h were measured. (E) Number of neutrophils in muscle 6 h after mRNA-LNP vaccination was assessed using flow cytometry. (A–D) *n* = 7, (E) *n* = 7–9, and (F) *n* = 6–33. (A–D) Data represent pooled results of two independent experiments. (E) Data represent pooled results of three independent experiments. (F) Data represent pooled results of eight independent experiments. (A–F) Data are expressed as means ± SD; ns, not significant by (A–D) Student’s *t* test or (E and F) Dunnett’s multiple comparison test vs. control.

Next, we attempted to elucidate the mechanisms underlying local adverse reactions. First, to assess whether neutrophils infiltrating the muscle increased vascular permeability at the vaccination site, Ly6G^+^ neutrophils were depleted using antibodies (Figure S9A), and the vascular permeability of the muscle was assessed. Neutrophil depletion did not alter the mRNA-LNP-induced increase in vascular permeability of the muscle (Figure 4A), suggesting that the infiltration of neutrophils contributes little to increased vascular permeability. In addition, the effects of neutrophils on systemic adverse reactions, such as fever, weight loss, and spontaneous behavior, were evaluated. Neutrophils did not affect the systemic adverse reactions (Figures 4B–4D and S9B). Next, we evaluated the possibility of IL-6, TNF-α, IFNAR-1, IL-1α, IL-1β, and IFN-γ contributing to local adverse reactions. Neutralizing antibodies against any of the cytokines did not influence the mRNA-LNP-induced increase in neutrophil infiltration or vascular permeability in the muscle (Figures 4E and 4F). These results indicated that local adverse reactions at the vaccination site were caused by a mechanism different from that of systemic adverse reactions.

### Effects of cytokines on immune responses

**Figure 5:**
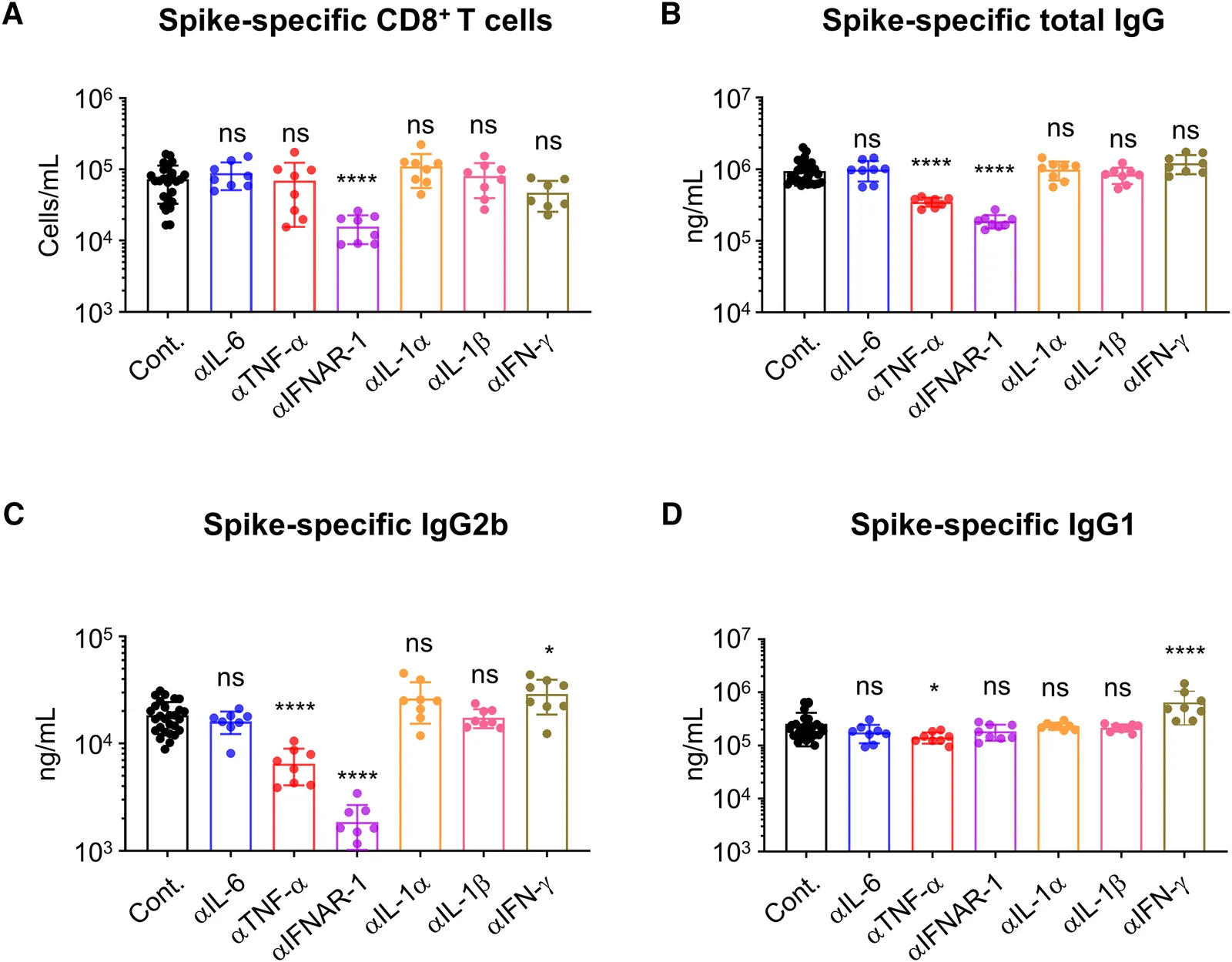
Effects of cytokines on antigen-specific immune responses Mice were administered neutralizing antibody 24 h before intramuscular vaccination with 20 μg mRNA-LNPs. (A) Number of spike-specific tetramer^+^ CD8^+^ T cells in the blood was examined 10 days after mRNA-LNP vaccination using flow cytometry. (B–D) Concentrations of spike-specific (B) IgG, (C) IgG2b, and (D) IgG1 in the plasma were assessed 14 days after mRNA-LNP vaccination using ELISA. (A–D) *n* = 7–28. Data represent pooled results of seven independent experiments. Data are expressed as means ± SD; ∗*p* < 0.05 and ∗∗∗∗*p* < 0.0001 by Dunnett’s multiple comparison test vs. control. ns, not significant.

For developing mRNA vaccines with reduced adverse reactions, it is important to verify antigen-specific immune responses when cytokines that induce adverse reactions are suppressed. Therefore, we evaluated the impact of cytokine inhibition on immune responses induced by the mRNA vaccine by measuring the number of spike-specific CD8^+^ T cells and spike-specific IgG concentrations (Figures 5 and S10). The neutralizing antibody against IFNAR-1 significantly reduced the number of CD8^+^ T cells (Figure 5A), whereas those against other cytokines did not induce significant changes (Figure 5A). At antigen-specific IgG concentrations, neutralizing antibodies against TNF-α and IFNAR-1 significantly decreased the total IgG and IgG2b levels (Figures 5B and 5C), and neutralization of TNF-α led to a modest, but statistically significant, decrease in IgG1 levels (Figure 5D). Furthermore, neutralizing antibodies against IL-6, IL-1α, IL-1β, and IFN-γ had no significant effect on CD8^+^ T cells or IgG concentrations, although neutralizing antibody against IFN-γ significantly enhanced the production of IgG2b and IgG1 (Figures 5A–5D). In particular, IL-6, which strongly induced systemic adverse reactions, did not significantly affect the induction of antigen-specific immune responses (Figures 5A–5D). These results indicated that initial transient inhibition of IL-6 could suppress systemic adverse reactions while maintaining the antigen-specific immune responses.

### Exploring mechanisms underlying the exacerbation of adverse reactions after the boost vaccination

**Figure 6:**
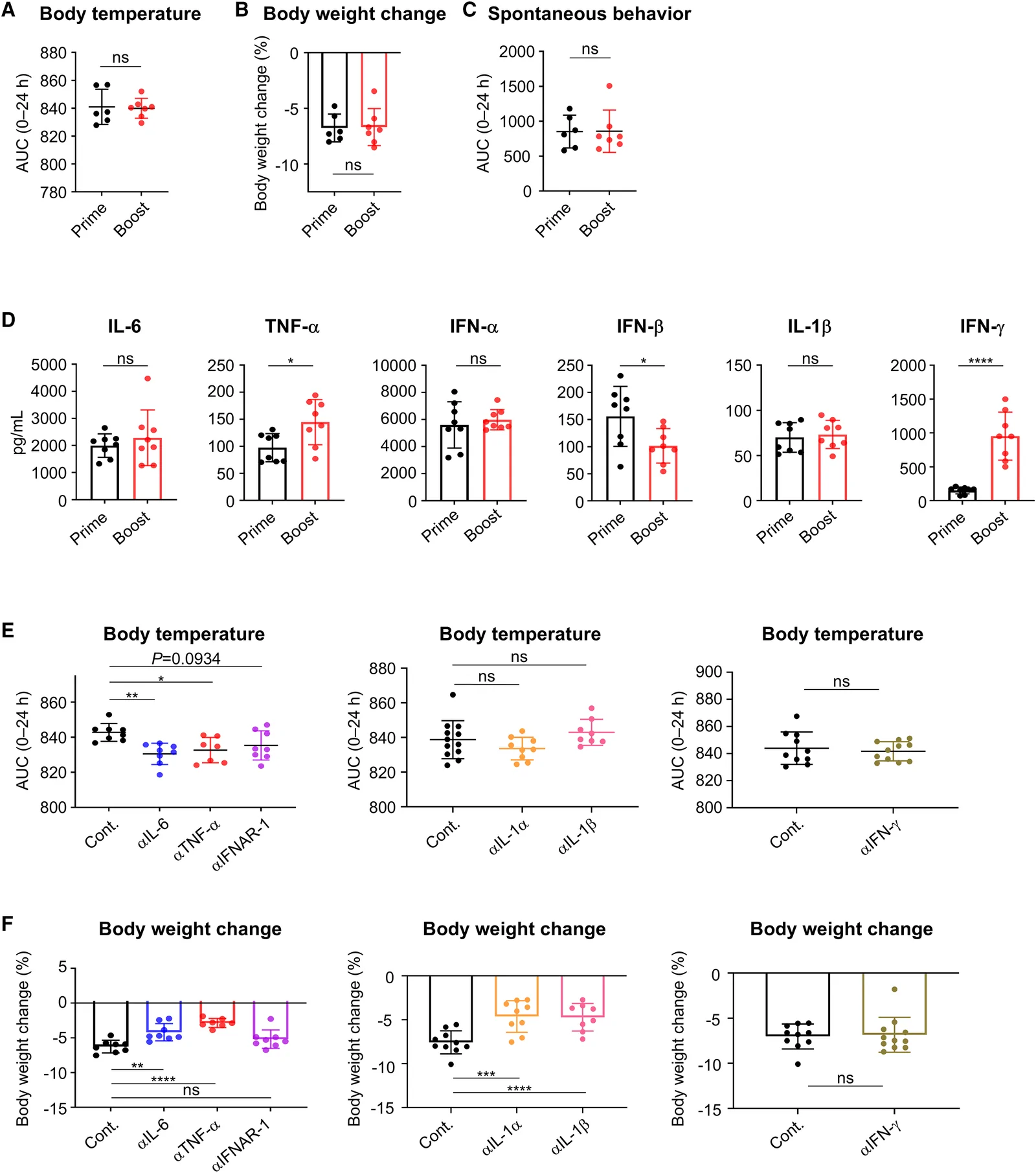
Mechanisms of exacerbation of adverse reactions after the boost vaccination Mice were vaccinated intramuscularly with 20 μg mRNA-LNPs on days 0 and 21. After vaccination, (A) area under the curve (AUC) for body temperature change, (B) body weight changes, and (C) AUC of behavior change over 24 h were measured. (D) Concentrations of IL-6, TNF-α, IFN-α, IFN-β, IL-1β, and IFN-γ in the plasma were assessed 6 h after the prime and boost vaccinations. (E and F) Mice were administered neutralizing antibody 24 h before the boost mRNA-LNP vaccination. After vaccination, (E) AUC for body temperature change and (F) body weight changes over 24 h were measured. (A–C) *n* = 6–7, (D) *n* = 8, and (E and F) *n* = 7–11. (A–F) Data are pooled results of two or three independent experiments. (E and F) Because of the experimental grouping, some data for the control groups are shared between the central and the right graphs. (A–F) Data are expressed as means ± SD; ∗*p* < 0.05, ∗∗*p* < 0.01, ∗∗∗*p* < 0.001, and ∗∗∗∗*p* < 0.0001 by Student’s *t* test in (A)–(D) and in (E) and (F) (in IFN-γ experiments) or by Dunnett’s multiple comparison test vs. control in (E) and (F) (except for IFN-γ experiments). ns, not significant.

To analyze the mechanism by which the frequency and severity of systemic adverse reactions increase after boost vaccination in humans, we first evaluated whether adverse reactions were exacerbated by the boost vaccination in mice. While adverse reactions were induced as in the prime vaccination for any of the endpoints (fever, body weight loss, and spontaneous behavior), we did not observe aggravation of adverse reactions compared with those after the prime vaccination (Figures 6A–6C and S11A). Next, focusing on cytokines important for inducing systemic adverse reactions after the prime vaccination, we compared blood cytokine levels at 6 h after the prime and boost vaccinations. Blood levels of TNF-α and IFN-γ were significantly elevated after the boost vaccination compared with those after the prime vaccination (Figure 6D). In contrast, the levels of other cytokines, such as IL-6, IFN-α, IFN-β, and IL-1β, showed no significant increase between the prime and the boost vaccinations (Figure 6D).

The levels of several cytokines, such as TNF-α and IFN-γ, were altered after the boost vaccination compared with those after the prime vaccination, suggesting that cytokines affecting systemic adverse reactions may differ between the prime and the boost vaccinations. Therefore, we examined the effects of cytokines on systemic adverse reactions to the boost vaccination. Neutralizing antibodies against IL-6 significantly suppressed fever and body weight loss, similar to the prime vaccination (Figures 6E, 6F, and S11B). In addition, neutralizing antibodies against IFNAR-1 showed a decreasing trend (*p* = 0.0954) for fever, but no change in body weight loss (Figures 6E, 6F, and S11B). Neutralizing antibodies against IL-1α and IL-1β significantly suppressed the body weight change (Figure 6F), although no significant change in fever was observed (Figures 6E and S11C). Furthermore, neutralizing antibody against TNF-α, the levels of which in the blood increased after the boost vaccination compared with those after the prime vaccination, suppressed body weight loss, as observed after the prime vaccination (Figure 6F). Fever was also significantly suppressed by neutralizing antibody against TNF-α after the boost vaccination (Figures 6E and S11B), although no significant difference was observed after the prime vaccination (Figure 3A). Although IFN-γ levels increased after the boost vaccination, neutralizing antibody against IFN-γ had no effect on fever or body weight loss (Figures 6E, 6F, and S11D). Neutralizing antibody against IFN-γ did not reduce antigen-specific IgG levels, although it significantly enhanced IgG2b production (Figure S12). In addition, spontaneous activity was not significantly changed in most neutralizing antibody-treated groups, whereas a significant increase was observed in mice treated with neutralizing antibody against IL-1β (Figure S13). Collectively, these results indicated that the levels of cytokines affecting systemic adverse reactions are almost similar between the prime and the boost vaccinations and that TNF-α appears to have a greater role in fever after the boost vaccination.

### Mechanisms of increase in TNF-α levels at the boost vaccination

**Figure 7:**
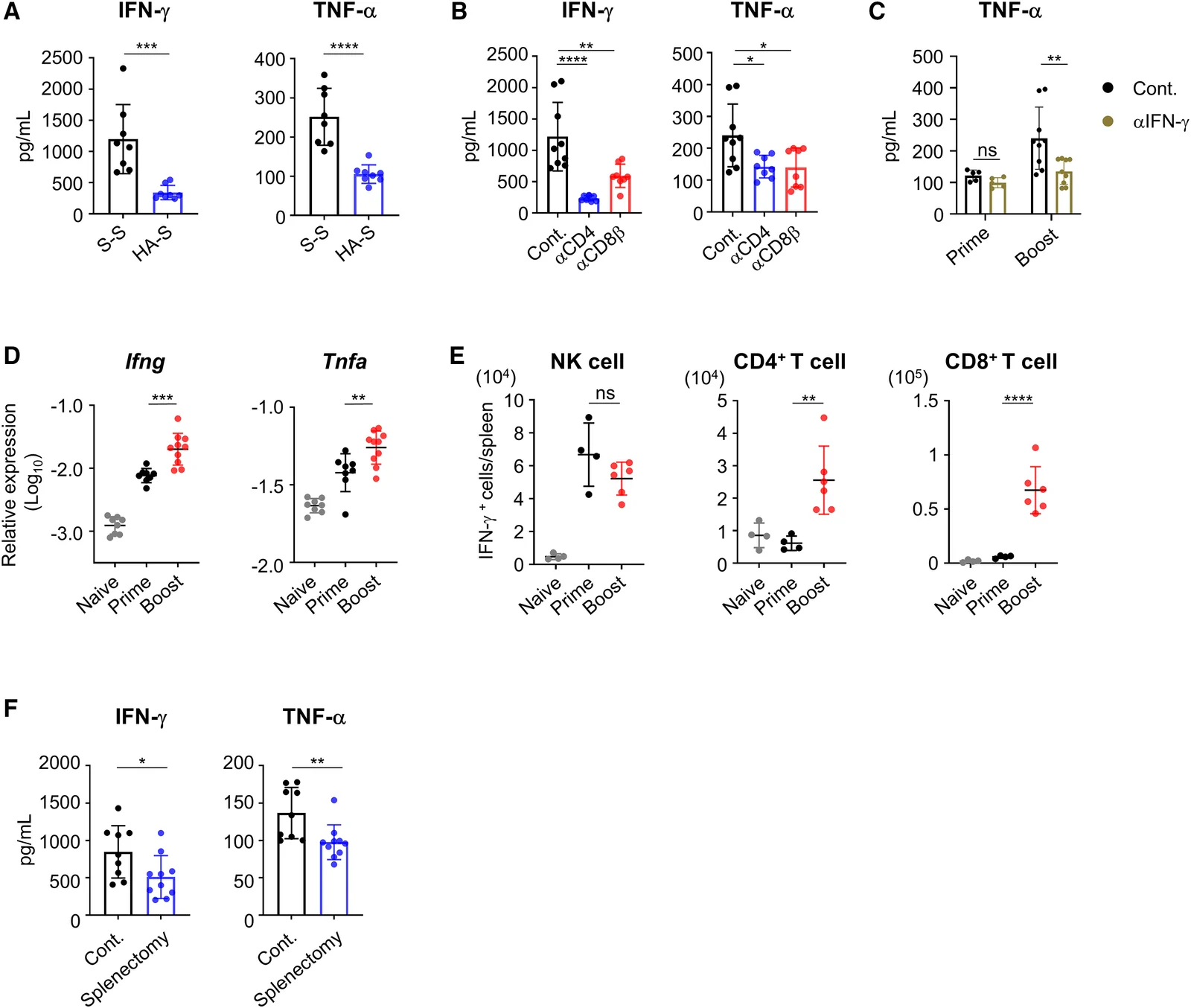
Mechanisms of increase in IFN-γ and TNF-α levels (A) Mice were vaccinated intramuscularly with 20 μg mRNA-LNPs encoding spike or HA protein on day 0, and all mice were vaccinated intramuscularly with 20 μg mRNA-LNPs encoding spike protein on day 21. IFN-γ and TNF-α levels in plasma were assessed 6 h after the boost vaccination. (B–F) Mice were vaccinated intramuscularly with 20 μg mRNA-LNPs on day 0 and day 21. (B) Mice were administered anti-CD4 or anti-CD8 antibody 24 h before the boost vaccination. IFN-γ and TNF-α levels in plasma were assessed 6 h after the boost vaccination. (C) Mice were administered anti-IFN-γ antibody 24 h before the prime or boost vaccination. TNF-α levels in plasma were assessed 6 h after the prime and boost vaccinations. Data for the prime and boost vaccinations were obtained independently and are presented together in the same plot. Data for the boost vaccination were acquired concurrently with the data presented in the panel on the right in (B). Therefore, the control data for the boost vaccination are the same as the control data in the plot on the right in (B). (D) Relative mRNA levels of *Ifng* and *Tnfa*, normalized to those of *Gapdh*, in the spleen were quantified 6 h after the prime and boost vaccinations using real-time PCR. (E) Numbers of IFN-γ^+^ NK, CD4^+^ T, and CD8^+^ T cells in the spleen 6 h after the prime and boost vaccinations were assessed using flow cytometry. (F) Mice were splenectomized 3 weeks before the prime mRNA-LNP vaccination. IFN-γ and TNF-α levels in plasma were assessed 6 h after the boost vaccination. (A) *n* = 8, (B and C) *n* = 5–9, (D) *n* = 8–10, (E) *n* = 4–6, and (F) *n* = 9–10. (A, B, D, and F) Data represent pooled results from two independent experiments. (C) For boost-vaccination-related experiments, data are pooled results from two or three independent experiments. (A–F) Data are expressed as means ± SD; ∗*p* < 0.05, ∗∗*p* < 0.01, ∗∗∗*p* < 0.001, and ∗∗∗∗*p* < 0.0001 by (A, C, and F) Student’s *t* test or (B, D, and E) Dunnett’s multiple comparison test vs. (B) control or (D and E) prime. ns, not significant.

We focused on acquired immunity to elucidate why TNF-α levels are increased after the boost vaccination. In addition to TNF-α, we also focused on IFN-γ, levels of which were significantly increased after the boost vaccination. Thus, to evaluate whether IFN-γ and TNF-α levels are enhanced by antigen-specific acquired immunity during the boost vaccination, we administered mRNA-LNPs encoding different antigens at the prime and boost vaccinations and measured blood cytokine levels after the boost vaccination. In addition to the spike protein, we used mRNA encoding the hemagglutinin (HA) protein of the H1N1 influenza virus. Blood IFN-γ levels did not differ significantly between the spike and the HA groups 6 h after the prime vaccination, whereas TNF-α levels exhibited a minor, but statistically significant, difference (Figure S14). However, the levels of IFN-γ and TNF-α were significantly lower when mRNA-LNP encoding different antigens (HA for prime and spike for boost) were administered compared with those encoding the same antigen (spike for prime and spike for boost) during the boost vaccination (Figure 7A). These results indicated that spike-specific acquired immunity plays an important role in enhancing IFN-γ and TNF-α levels after the boost vaccination.

We further focused on T cells, which are important components of acquired immunity. To assess the contribution of T cells in increasing IFN-γ and TNF-α levels, we administered antibodies to deplete CD4^+^ and CD8^+^ T cells 1 day before the boost vaccination (Figure S15) and measured the levels of IFN-γ and TNF-α in the blood 6 h after the boost vaccination. The levels of IFN-γ and TNF-α were significantly reduced upon depletion of either CD4^+^ or CD8^+^ T cells (Figure 7B). IFN-γ, produced by T cells, activates immune cells after boost vaccination with mRNA-LNPs.^20^ Therefore, it is possible that the elevated IFN-γ levels after the boost vaccination induce TNF-α production. We then administered a neutralizing antibody against IFN-γ and evaluated the effect on TNF-α. The neutralizing antibody significantly decreased TNF-α levels after the boost vaccination but had no significant effect after the prime vaccination (Figure 7C). Moreover, the neutralizing antibody against IFN-γ did not completely suppress TNF-α levels but only reduced the amount that was increased after the boost vaccination (Figure 7C). To characterize the cell populations responsible for TNF-α production after boost vaccination, splenocytes were isolated 3 weeks after the prime vaccination and stimulated *ex vivo* with spike protein. Intracellular staining for TNF-α was then performed to identify the TNF-α-producing cells. Following stimulation, B cells, macrophages, neutrophils, CD4^+^ T cells, and CD8^+^ T cells were found to produce TNF-α (Figures S16 and S17). To examine the involvement of IFN-γ in TNF-α production in this experiment, splenocytes were stimulated with spike protein in the presence of an anti-IFN-γ antibody. The production of TNF-α by macrophages, CD4^+^ T cells, and CD8^+^ T cells was unaffected by the neutralization of IFN-γ (Figure S17). In contrast, the abundance of TNF-α-producing B cells and neutrophils was significantly reduced compared with that in the isotype control group (Figure S17). Collectively, these findings indicated that IFN-γ produced by CD4^+^ T and CD8^+^ T cells induced an increase in TNF-α levels after the boost vaccination and that this effect of IFN-γ on TNF-α was boost specific.

As T cells are primarily located in secondary lymphoid tissues, these tissues may play a role in the induction of IFN-γ by T cells. We evaluated mRNA-LNP kinetics using mRNA encoding luciferase and observed antigen expression in the secondary lymphoid tissue, muscle, and liver (Figure S18). Therefore, it is possible that T cells are activated in the secondary lymphoid tissues and produce IFN-γ. Comparison of IFN-γ and TNF-α mRNA levels in the spleen showed that, similar to blood cytokine levels, the levels of these cytokines were higher after the boost than after the prime vaccination (Figure 7D). Next, to examine whether splenic CD4^+^ and CD8^+^ T cells directly produce IFN-γ after the boost vaccination, we evaluated splenic T cells 6 h after the prime and boost vaccinations by intracellular staining for IFN-γ (Figure S19). Natural killer (NK) cells produced IFN-γ after both the prime and the boost vaccinations, with no difference between the two (Figure 7E). Although the number of IFN-γ-producing CD4^+^ and CD8^+^ T cells after the prime vaccination was the same as in untreated mice, their numbers after the boost vaccination were significantly increased compared with those after the prime vaccination (Figure 7E). Finally, we performed splenectomy and evaluated its effect on the blood levels of IFN-γ and TNF-α after vaccination. Splenectomy significantly reduced IFN-γ and TNF-α levels after the boost vaccination (Figure 7F), whereas it did not affect cytokine production after the prime vaccination (Figure S20). Collectively, these results indicated that CD4^+^ and CD8^+^ T cells in the spleen are partially responsible for the increase in TNF-α levels via IFN-γ production after the boost vaccination.

## Discussion

In the present study, we elucidated the mechanisms underlying the induction of adverse reactions associated with mRNA vaccines. We found that the mRNA vaccine induced systemic adverse reactions, such as fever, body weight loss, and decreased spontaneous behavior, and local adverse reactions, such as increased vascular permeability and neutrophil infiltration in the muscles of mice. Moreover, empty LNPs without mRNA induced systemic and local adverse effects. LNPs induce cytokine production via activation of the NLR family pyrin domain containing 3 (NLRP3) inflammasome,^25^^,^^26^ MyD88, and TLR4 signaling^23^ in human peripheral blood mononuclear cells and in mice, whereas they exhibit adjuvant activity and are important in inducing immune responses.^27^ LNPs are generally composed of ionizable lipids, cholesterol, PEG-conjugated lipids, and phospholipids. Ionizable lipids are particularly important for LNP-induced inflammatory responses and adjuvant activity and have emerged as principal targets for optimizing LNP formulation.^28^^,^^29^ In contrast, the immunological effects of cholesterol, PEG-conjugated lipids, and phospholipids remain relatively underexplored compared with those of ionizable lipids. However, our recent findings indicate that other constituents of LNPs—particularly the phospholipid DSPC—also exert a significant influence on their inflammatory properties.^30^ Thus, inflammatory nature of LNPs may not be attributable solely to ionizable lipids, and future analyses should consider the potential contributions of additional structural components such as phospholipids. Collectively, these findings, including our results, suggest that the main cause of adverse reactions is the inflammatory nature of LNPs, especially ionizable lipids.^31^ However, empty LNPs without mRNA induced lower levels of IL-6 and IL-1β, which are important for the induction of systemic adverse reactions, than did mRNA-LNPs. In addition, type 1 IFN production was not induced. The mRNA in mRNA-LNPs activates TLR7/8, RIG-1, and MDA-5 signaling in human cells and mice.^20^^,^^32^ In addition, other reports have shown that mRNA in mRNA-LNPs especially induces IFN-β and activates dendritic cells.^33^ Therefore, mRNA may be involved in the induction of adverse reactions. The details of the contributions of LNPs and mRNA to the induction of adverse reactions need to be verified in the future.

We found that IL-6, TNF-α, type 1 IFN, and IL-1α especially contribute to fever and body weight loss after the prime vaccination. In particular, IL-6 had a significant effect on fever and weight loss. Receptors for IL-6 are expressed on cerebral vascular endothelial cells.^24^ The binding of IL-6 to their receptors induces COX-2, which in turn produces PGE2.^24^ Indeed, our findings that *Cox2* mRNA levels in the brain increased after mRNA-LNP vaccination and that fever was suppressed by ibuprofen, a COX-2 inhibitor, support this notion. Cytokines, such as IL-6 and TNF-α, are induced in the blood after mRNA vaccination, and the levels of IL-6 correlate with those of TNF-α in humans.^14^^,^^17^^,^^18^^,^^34^ Furthermore, IL-6 and TNF-α levels in blood after the boost vaccination of mRNA vaccine correlated strongly with systemic adverse reactions such as fever in humans.^14^^,^^17^^,^^18^ Considering these results in humans along with our findings, IL-6 and TNF-α may contribute to fever induction by mRNA vaccines in both humans and mice.

We found that body weight loss was strongly correlated with decreased food intake, which indicates that anorexia causes body weight loss. Anorexia has been observed under inflammatory conditions in mice, such as lipopolysaccharide (LPS) administration and influenza virus infection.^35^^,^^36^ These reports support our finding that many cytokines that contribute to fever also affect body weight loss. However, our results indicate that decreased spontaneous behavior is induced by a mechanism distinct from that responsible for fever. We administered mRNA-LNPs to the leg muscles of mice. The muscles at the injection site exhibited increased vascular permeability, which may be associated with pain. In fact, a previous study showed that mRNA-LNPs induce pain at the administration site in mouse legs.^37^ Thus, in our mouse model, leg pain may have prevented the mice from moving their legs, leading to a decrease in spontaneous behavior. Further studies are required to elucidate the underlying mechanisms.

Local adverse reactions were associated with increased vascular permeability and neutrophil infiltration in the muscles. We believe that cytokines that contribute to systemic adverse reactions might not affect these local reactions in the muscle. These results indicate that local and systemic adverse reactions are poorly correlated and occur via independent mechanisms, a notion consistent with previous reports showing no correlation between the severity of local and systemic adverse reactions following mRNA vaccination in humans.^14^ For vascular permeability, tight and adherens junctions maintain the integrity of vascular endothelial cells. Although inflammatory cytokines generally increase vascular permeability, our results show that cytokines, such as IL-6, TNF-α, type 1 IFN, IL-1α, IL-1β, and IFN-γ, do not contribute to mRNA-LNP-induced increase in vascular permeability. Direct action of LNPs on vascular endothelial cells may enhance their vascular permeability in a cytokine-independent manner. Several studies have shown that certain nanoparticles can open the endothelial paracellular route and induce endothelial leakiness, a phenomenon referred to as nanomaterial-induced endothelial leakiness (NanoEL).^38^^,^^39^ NanoEL is postulated to arise from physical interactions between exogenous nanoparticles and extracellular domains of vascular endothelial cadherin junctions.^38^^,^^39^ NanoEL-competent nanoparticles are typically anionic or almost neutral, with a size smaller than 100 nm.^38^^,^^39^ Thus, LNP-mediated increase in vascular permeability might be due to the induction of NanoEL by LNPs. Experiments using *in vitro* endothelial cell models aimed at assessing whether LNPs increase endothelial permeability could provide insights into the contribution of NanoEL to LNP-induced vascular leakiness. Further investigations, including those of the contribution of NanoEL, are needed to clarify the mechanism underlying increased vascular permeability.

We evaluated the effects of key cytokines, including IL-6, IL-1α, IL-1β, TNF-α, type 1 IFN, and IFN-γ, on the induction of antigen-specific CD8^+^ T cells and IgG. Inhibition of type 1 IFN signaling significantly reduced the induction of both CD8^+^ T cells and IgG. These results are consistent with those of a previous study using IFNAR-1-KO mice.^20^ In contrast, TNF-α was partially critical for IgG production without affecting the induction of CD8^+^ T cells. This observation aligns with previous reports on the effects of mRNA vaccines in TNF-α-inhibitor-treated patients with inflammatory bowel disease, suggesting that the inhibition of TNF-α negatively affects antibody induction while maintaining CD8^+^ T cell responses in humans.^40^ On the contrary, despite strongly inducing systemic adverse reactions, IL-6 did not significantly affect the induction of CD8^+^ T cells or IgG. While IL-6 has been reported to activate T follicular helper (Tfh) cells and promote germinal center (GC) formation in response to mRNA vaccines, our results contradict previous findings.^27^^,^^41^ This discrepancy may be due to differences in the timing of IL-6 inhibition. In the previous study, a neutralizing antibody against IL-6 was administered continuously after mRNA-LNP vaccination, and Tfh and GC activation were evaluated on day 5. In contrast, we administered the neutralizing antibody only once, 1 day before mRNA-LNP vaccination, which resulted in limited IL-6 inhibition. Therefore, we conclude that early inhibition of IL-6 is unlikely to affect the induction of CD8^+^ T cells and IgG. The inhibition of the expression and function of the cytokine could be a promising strategy for vaccine development to reduce adverse reactions without compromising vaccine effects. For example, systemic administration of anti-IL-6 antibodies to mitigate vaccine-related adverse reactions would be clinically unrealistic, as IL-6 plays an essential role in host defense against pathogens.^42^ In this regard, we previously reported the development of low-inflammatory LNPs with reduced inflammatory cytokine responses, including IL-6 secretion, and adverse reactions.^29^^,^^30^ We demonstrated that an mRNA-LNP vaccine containing a biodegradable ionizable lipid—SS-cleavable and pH-activated lipid-like material with oleic acid—induced lower levels of inflammatory cytokines and caused fewer adverse reactions than conventional mRNA-LNP.^29^ In addition, we developed LNPs in which the substitution of cholesterol with plant-derived sterols or the replacement of phospholipids with lipids possessing distinct head and tail group structures resulted in reduced production of inflammatory cytokines and milder adverse reactions.^30^ We propose that rational design of LNPs that minimizes excessive inflammatory cytokine responses, including IL-6, could be an optimal approach to developing mRNA-LNP vaccines with reduced adverse reactions.

We found that levels of TNF-α, which is important for systemic adverse reactions, such as body weight loss, were increased after the boost vaccination. Although TNF-α did not affect fever induced after the prime vaccination, it did so after the boost vaccination. Therefore, although the frequency and severity of systemic adverse reactions did not worsen after the boost vaccination, the increase in TNF-α levels might have contributed to the worsening adverse reactions after the boost vaccination. We found that IFN-γ produced by CD4^+^ and CD8^+^ T cells upon antigen restimulation after the boost vaccination may be crucial for this enhanced expression of TNF-α. Our results suggest that, upon boost vaccination, multiple cell types—including B cells, macrophages, neutrophils, CD4^+^ T cells, and CD8^+^ T cells—contribute to TNF-α production. Notably, TNF-α production by B cells and neutrophils appears to be IFN-γ dependent. Blood levels of IFN-γ and TNF-α were reported to increase following the boost injection of mRNA vaccine in humans,^14^^,^^17^^,^^34^^,^^43^ and the incidence and severity of adverse reactions strongly correlated with blood levels of IFN-γ and TNF-α.^17^^,^^18^ Moreover, previous reports have indicated that the rate of increase in IFN-γ-producing T cells in the blood during the boost vaccination correlates with a high frequency of adverse reactions.^44^ These observations in humans are consistent with those in mice, and it is highly likely that the TNF-α-induced exacerbation of adverse reactions observed in mice also occurs in humans.

We could reproduce adverse reactions, such as fever, reported in humans in a mouse model following prime and boost vaccinations. However, there are certain differences between mice and humans. IL-1 sensitivity has recently been reported to vary with the amount of IL-1 receptor antagonist (IL-1Ra) induced. IL-1Ra is an inhibitory protein that suppresses IL-1-receptor-mediated signaling and dampens inflammatory responses induced by IL-1. IL-1Ra is induced more strongly in mice than in humans after mRNA-LNP or mRNA-lipoplex vaccination,^32^ indicating that evaluating adverse reactions in mice may underestimate the inflammatory response induced by IL-1 in humans. Therefore, it is important to test the validity of the mechanism proposed in this study using IL-1Ra-KO mice. Additionally, our experimental system does not fully capture the exacerbation of adverse reactions observed in humans after the boost vaccination. In the present study, adverse reactions following boost vaccination were evaluated for a 20-μg-per-mouse dose. Reducing the vaccine dose may attenuate the fever observed after the prime vaccination and potentially allow the booster dose to elicit a more pronounced febrile response than the prime vaccination. In addition, the present study was conducted exclusively in female C57BL/6J mice. Systematic evaluation of differences related to the sex and strain of mice may further improve the model for mimicking human responses. Collectively, these approaches may contribute to the development of a mouse model that fully captures the exacerbation of adverse reactions observed in humans following booster vaccination. Moreover, sex differences in adverse reactions induced by mRNA vaccines have been reported in humans, with females reported to experience stronger adverse reactions than males.^45^^,^^46^ We observed these significant sex-related differences in our study. Therefore, this mouse model is expected to serve as a valuable tool for elucidating the mechanisms underlying sex-related differences in adverse reactions in humans. Furthermore, a comprehensive evaluation of both systemic and local adverse effects over an extended period of observation—rather than limited short-term assessments—would provide in-depth insights into vaccine safety. In particular, future studies should include pathological evaluation of tissue damage and behavioral assessments beyond spontaneous activity to better capture a broader spectrum of adverse reactions. Nevertheless, we believe that the proof of concept has been established in mice in our study, with relevant implications for humans. We believe that these findings provide fundamental insights into adverse reactions associated with mRNA vaccines in humans and may inform the development of mRNA vaccine formulations with reduced adverse reactions.

### Conclusion

This study showed that cytokines, such as IL-6, TNF-α, type I IFN, and IL-1, which are induced after the administration of mRNA vaccine, play critical roles in the induction of adverse reactions, such as fever and weight loss. Among these, IL-6 emerged as a key cytokine that strongly contributes to these adverse responses while exerting minimal impact on antigen-specific immune induction, which highlights its potential to reduce adverse reactions without compromising the effects of the vaccine. Furthermore, after the boost vaccination, increased TNF-α production—driven by T cells and IFN-γ—was found to exacerbate the adverse reactions. Our findings are consistent with clinical observations in humans and provide valuable insights for the development of next-generation mRNA-LNP vaccines with reduced reactogenicity.

## Materials and methods

### Study design

We did not use statistical methods to determine the sample size *a priori*. Randomization was used to assign mice to different treatment groups, and blinding was not performed because of practical limitations. Each experiment was performed more than twice, and experimental reproducibility was confirmed. All animal experiments were conducted in compliance with the ethical guidelines of The University of Osaka and were approved by the Animal Care and Use Committee of the Research Institute for Microbial Diseases and the Graduate School of Pharmaceutical Sciences at The University of Osaka, Japan (protocol numbers: BIKEN-AP-R01-15-2, BIKEN-AP-R02-14-6, and Douyaku R05-25).

### Mice

Female and male C57BL/6J mice (ages 6–8 weeks) were obtained from Oriental Yeast Co., Ltd (Kyoto, Japan). STING^−/−^ (RBRC09881) female mice (ages 7–14 weeks) were provided by RIKEN BRC through the National BioResource Project of MEXT/AMED (Tsukuba, Japan). Female wild-type C57BL/6J mice (ages 7–14 weeks) were acquired from CLEA (Shizuoka, Japan) for STING experiments. All mice were housed under a 12-h light/dark cycle and had unrestricted access to food and water. Mice in each group were housed in separate cages. Food intake was measured for each cage.

### Preparation of mRNA-LNP vaccine

Spike protein sequences were sourced from the SARS-CoV-2 virus (Wuhan-1 strain), and HA sequences were obtained from the influenza virus (A/California/07/2009). The cDNA for the spike protein incorporated a glycine substitution at position 614 (D614G), proline substitutions at positions 986 and 987 (K986P, V987P), and GSAS mutations at the furin cleavage site (R682G, R683S, and R685S). For transcription of mRNA expressing spike and HA, template pDNA was prepared using PCR. For firefly luciferase mRNA, the Positive Control Template in the kit was used as the template DNA. The pDNA was linearized with restriction enzymes, followed by phenol-chloroform extraction and ethanol precipitation. The linearized pDNA was transcribed into mRNA using the Takara IVTpro T7 mRNA Synthesis Kit (TAKARA Bio, Kusatsu, Japan), following the manufacturer’s instructions. N1-methylpseudouridine (Tokyo Chemical Industry, Tokyo, Japan) was incorporated by substituting uridine. A 5ʹ cap structure was added using CleanCap Reagent AG (TriLink BioTechnologies, San Diego, CA, USA) according to established protocol. The remaining double-stranded RNA was removed, as described previously.^47^ For formulating LNPs, a lipid mixture containing SM-102 (Cayman Chemical Company, Ann Arbor, MI, USA), DSPC (NOF Corporation, Tokyo, Japan), cholesterol (Sigma-Aldrich), and DMG-PEG_2000_ (NOF Corporation) was prepared in ethanol with the following composition: SM-102/DSPC/cholesterol/DMG-PEG_2000_ = 50/10/38.5/1.5. The mRNA-LNPs were generated by combining the mRNA with the lipid mixture in ethanol at an N/P ratio of 5.5, using the NanoAssemblr instrument (Precision Nanosystems, Vancouver, BC, Canada) at a 3:1 flow rate ratio. The external solution of the mRNA-LNPs was then exchanged with D-PBS (Nacalai Tesque, Kyoto, Japan) through ultrafiltration using Amicon Ultra-15-100K centrifugal units (Merck KGaA, Darmstadt, Germany). The size and zeta potential of the mRNA-LNPs were assessed using a Zetasizer Nano ZS (Malvern Instruments, Malvern, UK). Encapsulation efficiency was measured using the Ribogreen reagent (Invitrogen, Carlsbad, CA, USA). Empty LNPs were generated using the same procedure employed for the mRNA-LNPs, but omitting the mRNA.

### mRNA-LNP vaccination

Mice were administered 5, 10, or 20 μg of mRNA-LNPs expressing spike protein via the tibialis anterior muscle. The boost vaccination was administered 21 days after the prime vaccination. Five or 10 μg was administered to one leg, and 20 μg was administered to both legs (10 μg each). The dose was defined by the amount of mRNA.

### Measurement of body temperature and spontaneous behavior

Nanotag (KISSEI COMTEC, Nagano, Japan) was programmed to automatically monitor locomotor activity and body temperature every 5 min prior to surgery. The mice were anesthetized, and a longitudinal incision was made in the skin on their back. The subcutaneous tissue was then carefully detached to create space for the Nanotag. After Nanotag implantation, the skin was closed using a suture clip (Natsume Seisakusho, Osaka, Japan). One week after implantation, the mice were used for experiments. Nanotag contains a three-axis accelerometer and can count both horizontal (*x* and *y* axes) and vertical (*z* axis) activities. To start and stop data recording, we touched the radio frequency identification card reader (PaSoRi) on the implanted Nanotag. Hourly averages of locomotor activity and body temperature were calculated and used for the data analysis. The rectal body temperature was measured using an animal temperature recorder designed for mice (Natsume Seisakusho).

### Vascular permeability in muscle

Mice were administered 10 μg mRNA-LNP in the anterior tibialis muscle. At designated time points, the mice were intravenously administered Evans blue dye (BioLegend; 200 mg/kg). Thirty minutes post-administration, muscle samples were collected from the vaccination site, weighed, and incubated with 500 μL of formamide (Wako Pure Chemical Industries, Ltd., Osaka, Japan) at 55°C for 48 h to extract the dye. The optical density of the extracted dye was measured at 620 nm using a microplate reader and compared with a standard calibration curve generated using formamide (Wako) to quantify the Evans blue dye present.

### Inflammatory cells in muscle

Mice were administered 10 μg mRNA-LNPs in the anterior tibialis muscle, which was collected at 6 h after mRNA-LNP vaccination. The muscle tissue was then incubated in a solution containing 0.2% w/v collagenase type 2 (Worthington Industries, Columbus, OH, USA) and 200 U/mL DNase I (Roche Diagnosis GmbH, Mannheim, Germany) at 37°C with shaking at 200 rpm for 1.5 h. A single-cell suspension was prepared by filtering the tissue through a cell strainer (Corning, Inc., Corning, NY, USA). For surface antigen staining, the cells were incubated with Fixable Viability Dye eFluor 780 (Invitrogen) and antibodies. The antibodies used are listed in Table S1. The staining process was conducted in PBS supplemented with 2% FBS, 1 mM ethylenediaminetetraacetic acid (EDTA; DOJINDO, Kumamoto, Japan), and 0.05% sodium azide (Wako) for 20 min at 4°C in the dark. Flow cytometry was performed using an Attune NxT flow cytometer (Thermo Fisher Scientific, Waltham, MA, USA). The FlowJo software v.10.8.1 was used for analyses.

### Quantitation of cytokines and chemokines in blood

Mice were administered 20 μg of mRNA-LNPs, and plasma samples were prepared from blood collected after primary and boost vaccinations. Plasma concentrations of cytokines and chemokines were assessed using the LEGENDplex Mouse Anti-Virus Response Panel (BioLegend) according to the manufacturer’s instructions. Additionally, IL-1α levels were quantified using ELISA kits from BioLegend.

### Real-time quantitative reverse transcription PCR

Mice were administered 20 μg mRNA-LNPs and organs were collected after vaccination. RNA was extracted using TRIzol reagent (Invitrogen) and cDNA was synthesized via reverse transcription using the ReverTra Ace qPCR RT Master Mix with gDNA Remover (Toyobo Co., Ltd., Osaka, Japan). The levels of cytokine mRNAs in the anterior tibialis muscle, inguinal lymph nodes, spleen, and liver were evaluated using real-time PCR analysis on a Light Cycler 480 SYBR Green I Master (Thermo Fisher Scientific) with the primers listed in Table S2. Expression was normalized against *Gapdh* expression.

### Cytokine neutralization antibody and ibuprofen treatment

To neutralize cytokines, mice were intraperitoneally administered a single 200-μg dose of the anti-IL-6, anti-TNF-α, anti-IFNAR-1, anti-IL-1α, anti-IL-1β, or anti-IFN-γ antibody in 200 μL PBS 24 h before vaccination. Except where specified otherwise, no additional antibody doses were administered after vaccination. For example, in the experiment presented in Figure 5, mice received a single dose of a neutralizing antibody prior to vaccination. The number of spike-specific tetramer^+^ CD8^+^ T cells in the peripheral blood was determined 10 days after mRNA-LNP vaccination via flow cytometry, and spike-specific IgG, IgG1, and IgG2b levels in the plasma were assessed 14 days after vaccination using ELISA, as described below. In an experiment specifically designed to assess vascular permeability in muscle using Evans blue dye, an additional antibody dose was administered. In this experiment, 40 μg of antibody was added to mRNA-LNPs and administered intramuscularly, in addition to an intraperitoneal dose of 200 μg, 24 h before vaccination. The neutralizing antibodies are listed in Table S3. For Figures 3A–3C, PBS was used as a control for neutralizing antibodies against IL-6, TNF-α, and IFNAR-1, and the isotype control antibody was used as a control for neutralizing antibodies against IL-1α and IL-1β. The isotype control antibody was used as a control for neutralizing antibodies against IFN-γ. For Figures 6E and 6F, PBS was used as a control for neutralizing antibodies against IL-6, TNF-α, and IFNAR-1, and the isotype control antibody or PBS was used as a control for neutralizing antibodies against IL-1α, IL-1β, and IFN-γ. For the data illustrated in Figure 4E, PBS was used as a control for neutralizing antibodies. For the data illustrated in Figure 5, PBS or isotype control antibody was used as a control for neutralizing antibodies. For Figure 4F, PBS or the isotype control antibody was used as a control for the neutralizing antibody. In addition, ibuprofen (Nacalai Tesque; 400 μg) was administered intraperitoneally 30 min before and 6 h after mRNA-LNP vaccination.

### Depletion of Ly6G^+^ cells

To deplete Ly6G^+^ cells, mice were intraperitoneally administered an anti-Ly6G antibody (clone: 1A8; BioLegend). PBS or rat IgG2a isotype control (BioLegend) was used. Each antibody or PBS was intraperitoneally administered 2 days (300 μg) and 1 day (100 μg) before mRNA-LNP vaccination.

### Recombinant protein

The spike protein of SARS-CoV-2 (Wuhan-Hu-1) was produced in-house. The cDNA encoding the spike ectodomain (amino acids 1–1,208) was engineered to incorporate a glycine substitution at position 614 (D614G), proline substitutions at positions 986 and 987 (K986P and V987P), and a GSAS substitution at the furin cleavage site (R682G, R683S, and R685S). This was followed by addition of the bacteriophage T4 fibritin foldon sequence (GYIPEAPRDGQAYVRKDGEWVLLSTFL) and a C-terminal histidine tag (His-tag). The resulting construct was cloned into the pcDNA3.1 expression plasmid (Thermo Fisher Scientific). The expression vectors were then transfected into Expi293F cells according to the manufacturer’s instructions (Thermo Fisher Scientific). Four days after transfection, the cell culture supernatant was centrifuged to isolate the spike protein-containing supernatant. The spike protein was subsequently purified using an AKTA Explorer chromatography system employing a Ni-Sepharose HisTrap FF column (GE Healthcare, Chicago, IL, USA), followed by a Superose 6 Increase 10/300 GL column (GE Healthcare).

### Detection of antigen-specific antibodies

Mice were administered 20 μg mRNA-LNPs intramuscularly, and blood samples were collected 14 days after the prime vaccination and 9 days after the boost vaccination. The levels of spike-specific antibodies in plasma samples were evaluated using ELISA. Briefly, 1 μg/mL spike protein in 0.1 M sodium carbonate buffer (pH 9.6) was coated onto the wells of 96-well plates by incubating overnight at 4°C. The plates were treated with Block Ace (DS Pharma Biomedical, Osaka, Japan) to prevent nonspecific binding. Plasma samples were added to the antigen-coated wells. The antibodies listed in Table S4 were used for detection and quantification of antibodies. After additional washes, tetramethylbenzidine (Nacalai Tesque) was added to develop color, which was subsequently stopped using 2 N H_2_SO_4_. Absorbance was measured at 450–570 using a microplate reader (Power Wave HT, BioTek Instruments, Inc., Winooski, VT, USA).

### Quantitation of spike-specific CD8^+^ T cells in the blood using flow cytometry

Mice were administered 20 μg mRNA-LNPs intramuscularly, and blood samples were collected 10 days after the prime vaccination. Red blood cells were removed by lysing whole blood with a lysis buffer containing 155 mM NH_4_Cl and 10 mM Tris-HCl. For surface antigen staining, cells were incubated with Fixable Viability Dye eFluor 780 (Invitrogen), spike-specific tetramer H-2Kb/S539-546 (the biotinylated monomer was sourced from the NIH Tetramer Core Facility and tetramerized with APC-streptavidin from Agilent Technologies, Santa Clara, CA, USA), and antibodies. The antibodies used are listed in Table S1. This staining process was conducted in PBS supplemented with 2% FBS, 1 mM EDTA, and 0.05% sodium azide for 15 min at 37°C in the dark. Flow cytometry was performed using an Attune NxT flow cytometer (Thermo Fisher Scientific). The FlowJo software v.10.8.1 was used for the analysis.

### Luciferase expression

Mice were administered 20 μg mRNA-LNPs encoding luciferase. Six hours after the prime or boost vaccination, the muscle, draining lymph nodes, spleen, and liver were collected from euthanized mice. Each tissue sample was weighed and homogenized in PBS containing a protease inhibitor cocktail (Nacalai Tesque) using stainless-steel beads and a μT-12 bead crusher (Taitec Corporation, Shizuoka, Japan). Luciferase luminescence was assessed by treating the supernatant with the ONE-Glo Luciferase Assay System (Promega) for 5 min and taking the readings using the GloMax Discover Microplate Reader (Promega Corporation, Madison, WI, USA).

### Depletion of CD4^+^ and CD8^+^ T cells

To deplete CD4^+^ and CD8^+^ T cells, mice were intraperitoneally administered 100 μg anti-CD4 antibody (clone: GK1.5; Selleck Chemicals, Houston, TX, USA) or anti-CD8β antibody (clone: 53-5.8; Bio X Cell, West Lebanon, NH, USA) in PBS 1 day before the boost vaccination. PBS was used as a control for antibodies.

### Splenectomy

Splenectomy was performed under anesthesia 3 weeks prior to immunization, as described below. The abdomen was shaved and disinfected, and a lateral skin incision was made on the left side of the mouse to access the abdominal cavity. The vascular pedicles of the spleen were ligated using a silk suture (Nesco, Inc., Tokyo, Japan), after which the spleen was carefully separated from the surrounding tissues and fully removed. The skin and abdominal incisions were closed using a weak-curved surgical suture needle (Natsume Seisakusho) and suture clip (Natsume Seisakusho). This method ensured complete removal of the spleen, with no splenic tissue left behind, as confirmed during euthanasia. For sham surgery, an incision was made in the left flank without spleen removal.

### Quantitation of IFN-γ-producing cells in the spleen using flow cytometry

Mice were administered 20 μg mRNA-LNPs intramuscularly. The spleen was collected 6 h after the prime or boost vaccination and incubated in a solution containing 200 μg/mL Liberase TL (Roche Diagnosis GmbH), 10 U DNase I (Roche Diagnosis GmbH), and protein transport inhibitor cocktail (Thermo Fisher Scientific) for 20 min at 37°C. Red blood cells were removed by lysing whole blood with a lysis buffer containing 155 mM NH_4_Cl and 10 mM Tris-HCl. For surface antigen staining, cells were incubated with the Fixable Viability Dye eFluor 780 and antibodies. The antibodies used are listed in Table S1. This staining process was conducted in PBS supplemented with 2% FBS, 1 mM EDTA, and 0.05% sodium azide for 20 min at 4°C in the dark. Intracellular cytokine staining was performed using the BD Cytofix/Cytoperm Fixation/Permeabilization Kit (BD Biosciences, San Jose, CA, USA) following the manufacturer’s instructions. Flow cytometry was performed using an Attune NxT flow cytometer (Thermo Fisher Scientific). The FlowJo software v.10.8.1 was used for the analysis.

### Quantitation of TNF-α-producing cells in the spleen using flow cytometry

Mice were intramuscularly administered 20 μg mRNA-LNPs encoding spike. The spleens were collected 21 days after the prime vaccination. The splenocytes were treated with spike protein (50 μg/mL) in the absence or presence of the anti-mouse IFN-γ (100 μg/mL) antibody in RPMI 1640 medium containing 10% FBS, 1% penicillin-streptomycin, and 50 mM 2-mercaptoethanol at 37°C for 19 h. As a control for anti-mouse IFN-γ antibody, a rat IgG1 isotype control was used. The antibodies that were used are listed in Table S3. Subsequently, a protein transport inhibitor cocktail was added, and incubation was continued for an additional 5 h at 37°C in 96-well plates. For surface antigen staining, cells were incubated with the Fixable Viability Dye eFluor 780 and antibodies. The antibodies used are listed in Table S1. The staining was conducted in PBS supplemented with 2% FBS, 1 mM EDTA, and 0.05% sodium azide for 20 min at 4°C in the dark. Intracellular cytokine staining was performed using the BD Cytofix/Cytoperm Fixation/Permeabilization Kit following the manufacturer’s instructions. Flow cytometry was performed using an Attune NxT flow cytometer (Thermo Fisher Scientific). The FlowJo software v.10.8.1 was used for analysis.

### Statistical analyses

The GraphPad Prism software v.10.4.1 (GraphPad Software, San Diego, CA, USA) was used for statistical analyses. A two-tailed unpaired Student’s *t* test was performed for comparing between two groups, and one-way ANOVA followed by Dunnett’s and Tukey’s multiple comparison test was used for comparisons involving more than two groups. For data displayed in graphs with logarithmic axes, log transformation was performed prior to analysis. A *p* value of <0.05 was considered statistically significant. Data are presented as means ± standard deviation (SD).

### Data and code availability

All data are included in the paper or the supplemental information. Additional data are available from the corresponding authors on reasonable request.

## Acknowledgments

This study was supported by grants from the 10.13039/501100000646Japan Society for the Promotion of Science, Japan (JSPS KAKENHI grant numbers JP23K27343 and JP24K22020 to Y.Y.), the 10.13039/100009619Japan Agency for Medical Research and Development, Japan (AMED grant numbers 23am0401030h0003 and JP223fa627002 to Y.Y.), the All-Osaka U Research in “10.13039/501100022711The Nippon Foundation–Osaka University Project for Infectious Disease Project”, Japan (to Y.Y.), and 10.13039/501100019670The Research Foundation for Microbial Diseases of Osaka University, Japan (BIKEN to Y.Y.). The graphical abstract was created with BioRender.

## Author contributions

The manuscript was written with contributions of all authors. All authors have given approval to the final version of the manuscript.

## Declaration of interests

T.K., Y. Kunishima, and Y.Y. were employed by the Research Foundation for Microbial Diseases of Osaka University.

## Supplemental information

Document S1. Figures S1–S20 and Tables S1–S4

Document S2. Article plus supplemental information
