# Supplementary material for: Inflammatory mediators of mRNA vaccine-induced adverse reactions in mice
Source: Mol Ther. 2026 Jan 20;34(4):2496–512. doi: 10.1016/j.ymthe.2026.01.022 (PMC13069329; doi:10.1016/j.ymthe.2026.01.022)
Supplement: Supplementary file 1 [file mmc1.pdf]

YMTHE, Volume *34*

Supplemental Information

Inflammatory mediators of mRNA vaccine-induced adverse reactions in mice

Koyo Honda, Tatsuya Karaki, Yuta Kunishima, Yoshino Kawaguchi, Naoki Takemura, Takashi Matsuzaki, So-ichiro Fukada, Tatsuya Saitoh, Toshiro Hirai, and Yasuo Yoshioka

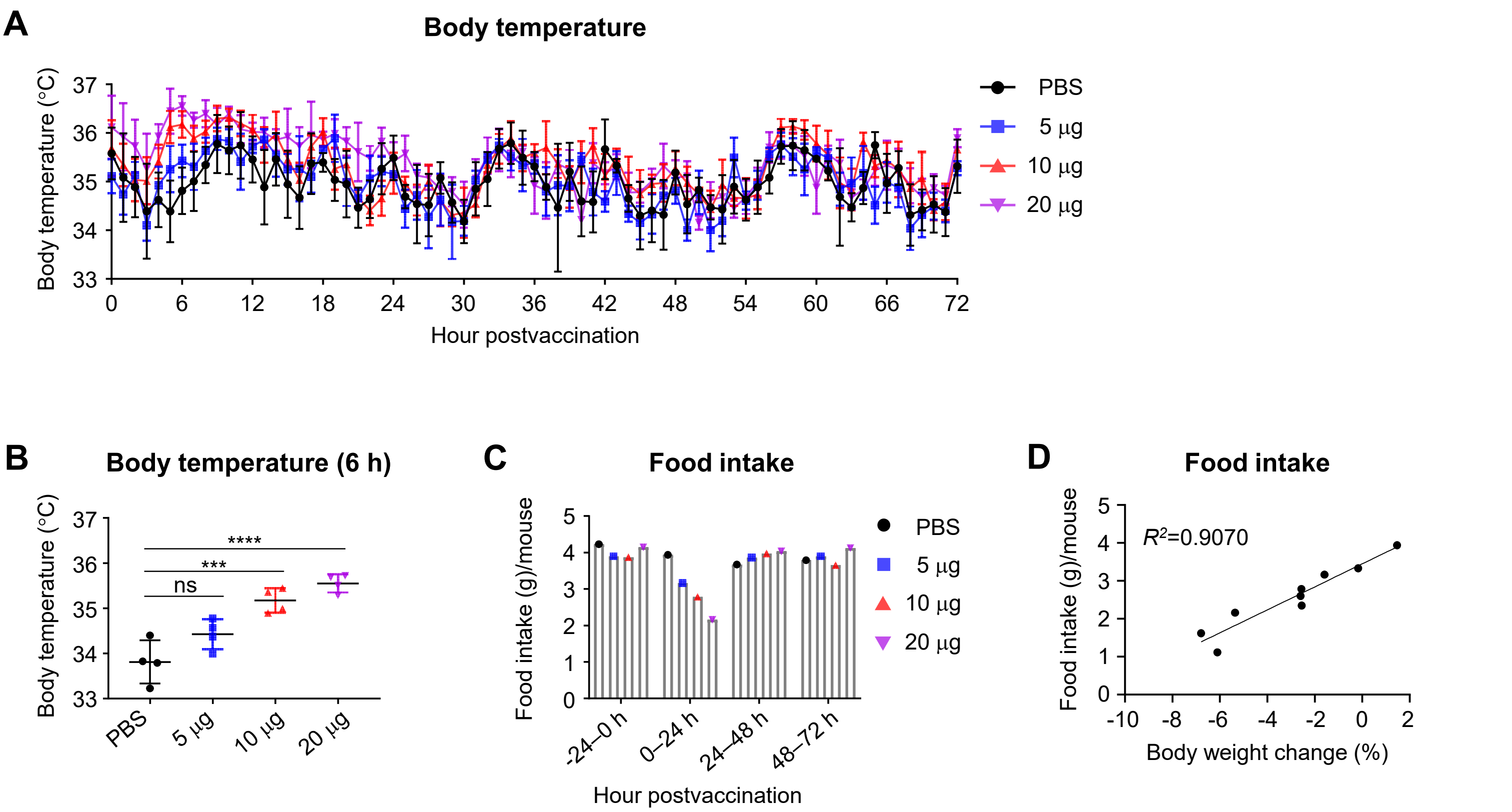

Figure S1. Dose-dependent effects of mRNA-LNP vaccination on body temperature and food intake in mice. (A-C) Mice were intramuscularly injected PBS or 5, 10, or 20 µg of mRNA-LNP. After injection, (A) body temperature from 0 to 72 h, (B) body temperature at 6 h, and (C) changes in average food intake per mouse in the same group every 24 h over 72 h were measured. (D) Mean changes in food intake and body weight over 24 h after the PBS or prime vaccination were plotted, and correlation between these parameters were evaluated. (A, B) n = 4, (C, D) n = 1. (A, B) Data are expressed as means ± SD; ***P<0.001; ****P<0.0001 by (B) Dunnett's multiple comparison test vs. PBS. ns, not significant.

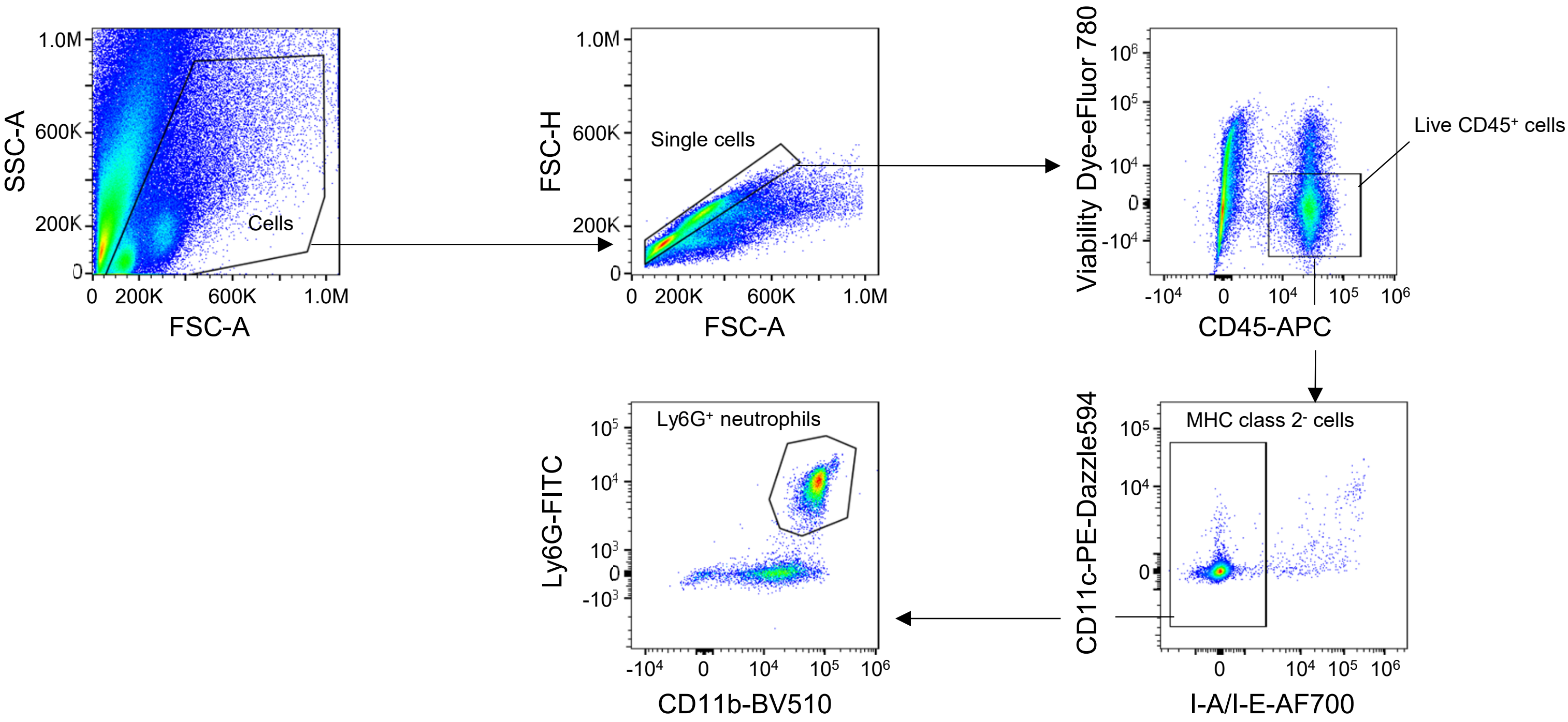

Figure S2. Gating strategy for neutrophils in muscle.
Gating strategy for neutrophils in the muscle after mRNA-LNP vaccination.

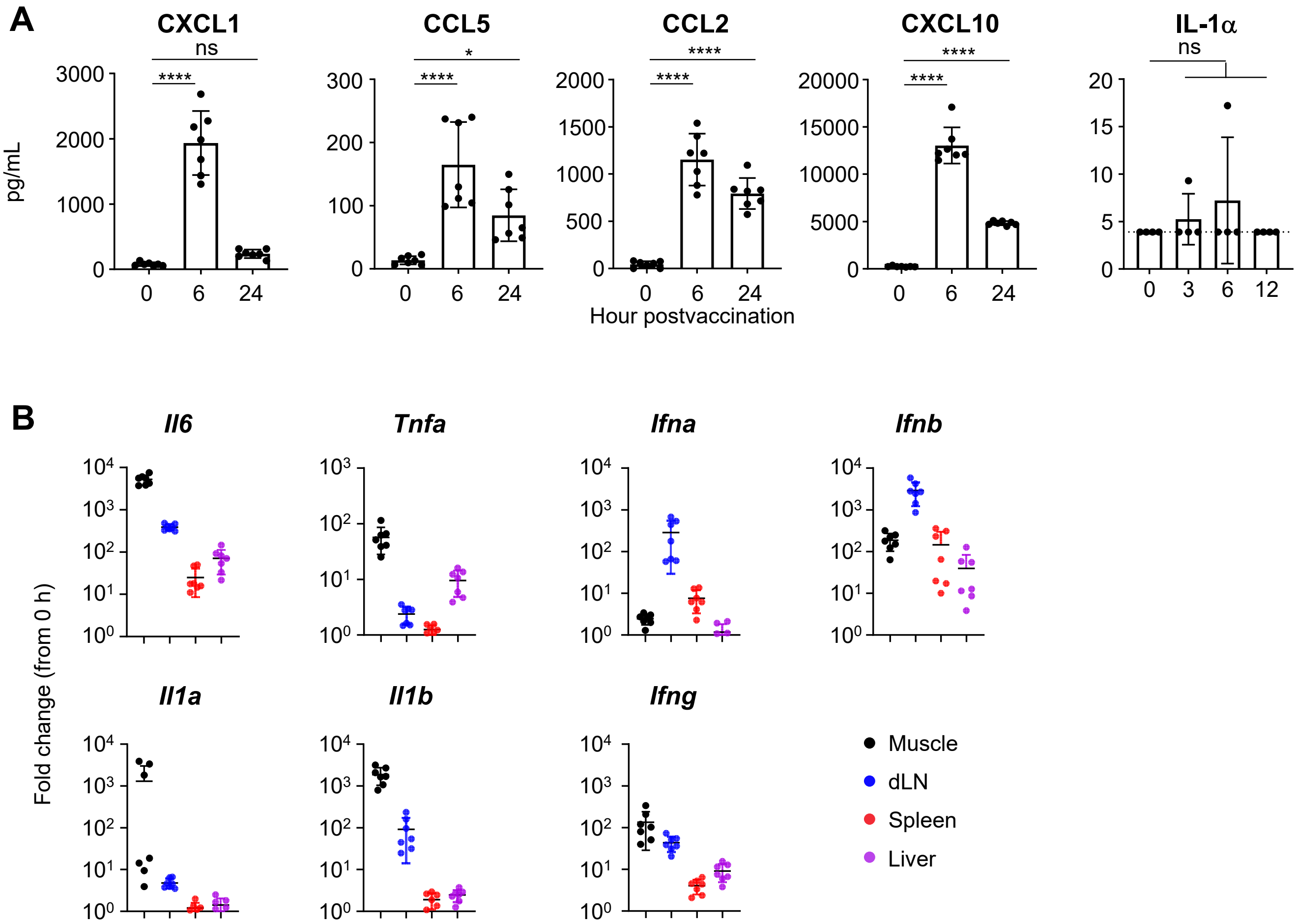

Figure S3. Temporal changes in the levels of cytokines.

(A, B) Mice were vaccinated intramuscularly with 20 μg mRNA-LNP. (A) Concentrations of CXCL1, CCL5, CCL2, CXCL10, and IL-1α in plasma were assessed for 0–24 h after mRNA-LNP vaccination. (B) Fold changes in mRNA levels of *Il6*, *Tnfa*, *Ifna*, *Ifnb*, *Il1a*, *Il1b*, and *Ifng*, normalized to *Gapdh*, in tissue were quantified 6 h after mRNA-LNP vaccination using real-time PCR. The detection limit was set at a Cp value of 40 cycles. (A, B) n = 7. (A) In the IL-1α panel, the dotted line represents the detection limit. Data are pooled results of two independent experiments excepting for IL-1α experiment in (A). (A, B) Data are expressed as means ± SD. (A) *P<0.05, ****P<0.0001; by Dunnett's multiple comparison test vs. 0 h. ns, not significant.

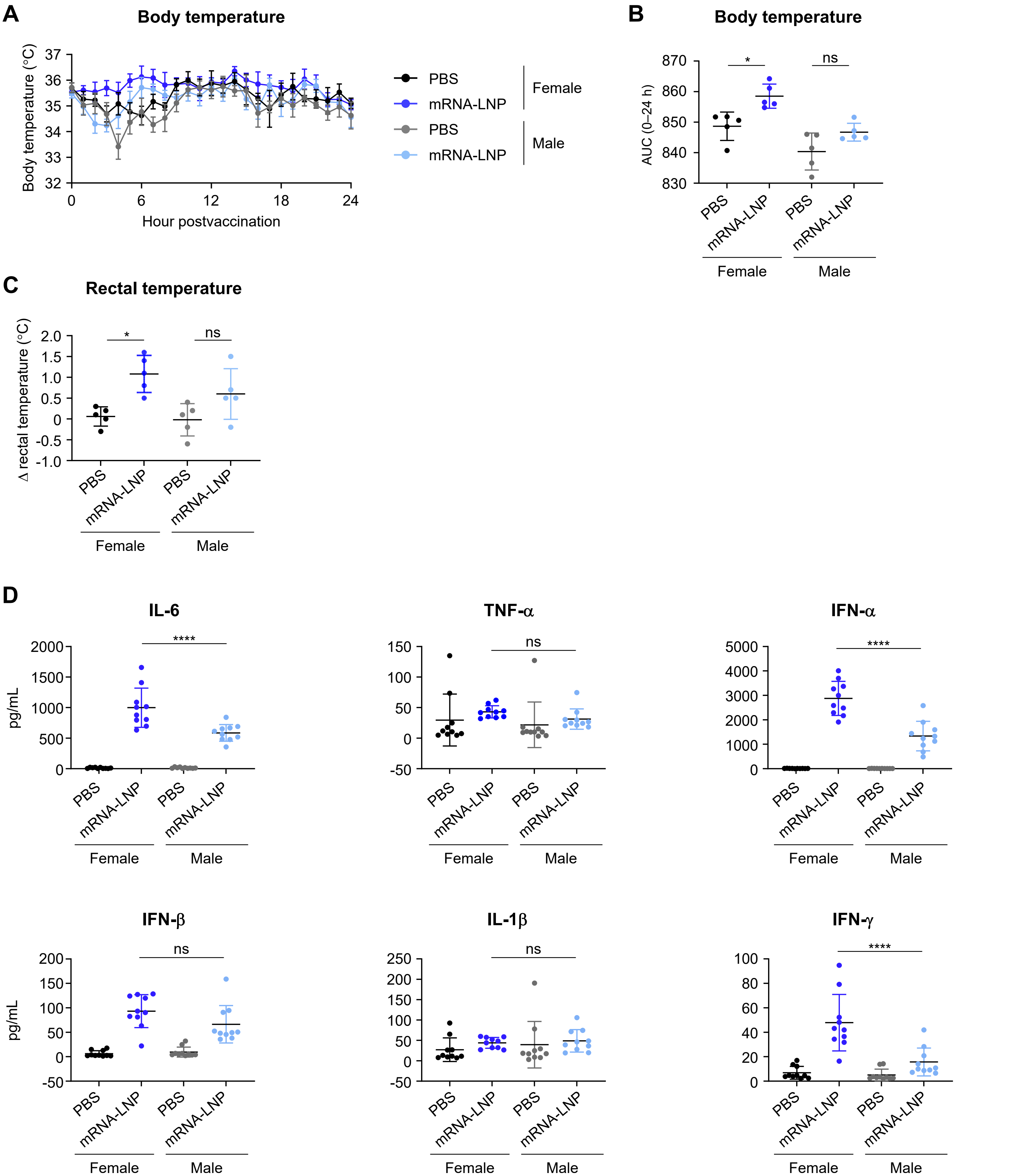

Figure S4. Differences in adverse reactions between female and male mice.
Female and male mice were vaccinated intramuscularly with 20 μg mRNA-LNP. (A) The body temperature and (B) area under the curve (AUC) for body temperature change were measured over 24 h. (C) The rectal body temperature was measured at 6 h after mRNA-LNP vaccination. (D) Plasma levels of cytokines were measured at 6 h after mRNA-LNP vaccination. (A-C) *n* =5, (D) n =10. (D) Data are pooled results of two independent experiments. (B-D) Data are expressed as means ± SD; *P<0.05, ****P<0.0001; by Student's *t*-test and Tukey's multiple comparison test. ns, not significant.

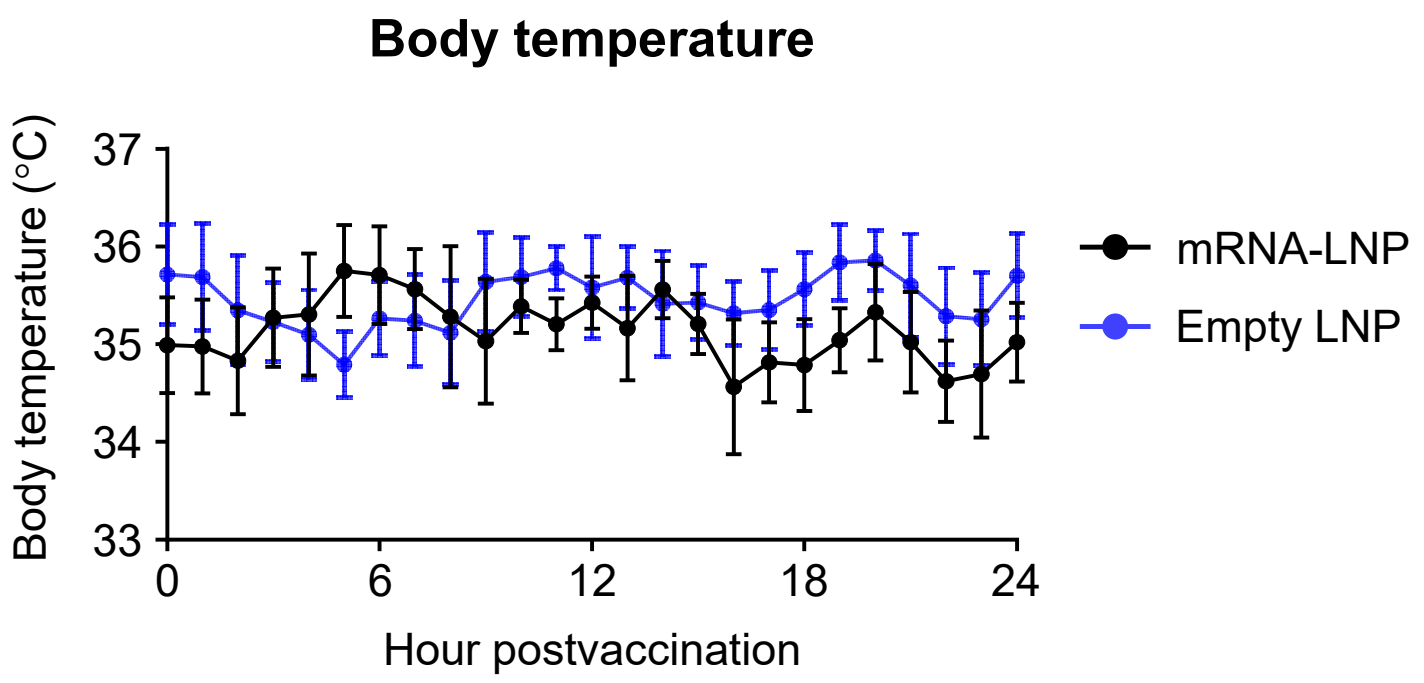

Figure S5. Effect of LNP on the induction of adverse reactions.
Mice were vaccinated intramuscularly with 20 μg mRNA-LNP or empty LNP without mRNA. After vaccination, the body temperature was measured over 24 h. n = 8–10. Data are pooled results of two independent experiments. Data are expressed as means ± SD.

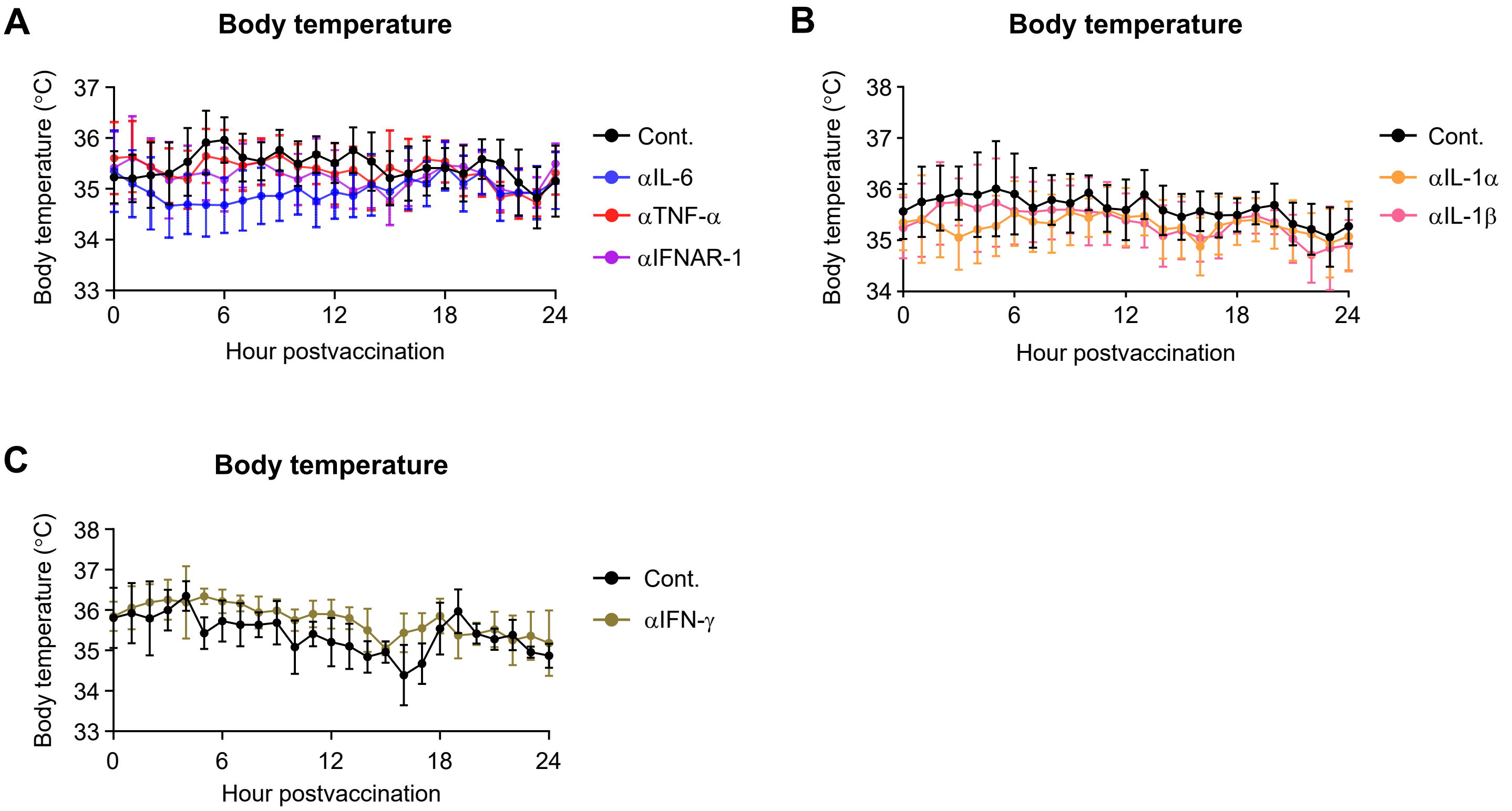

Figure S6. Effect of cytokines on the induction of adverse reactions after the prime vaccination.
Mice were administered a neutralizing antibody 24 h before intramuscular vaccination with 20 μg mRNA-LNP. After vaccination, body temperature was measured over 24 h. (A) PBS was used as a control for neutralizing antibody. (B, C) Isotype control antibody was used as a control for neutralizing antibody. (A) n = 13–14, (B) n = 11, (C) n =5–6. (A-C) Data are pooled results of two or three independent experiments. (A-C) Data are expressed as means ± SD.

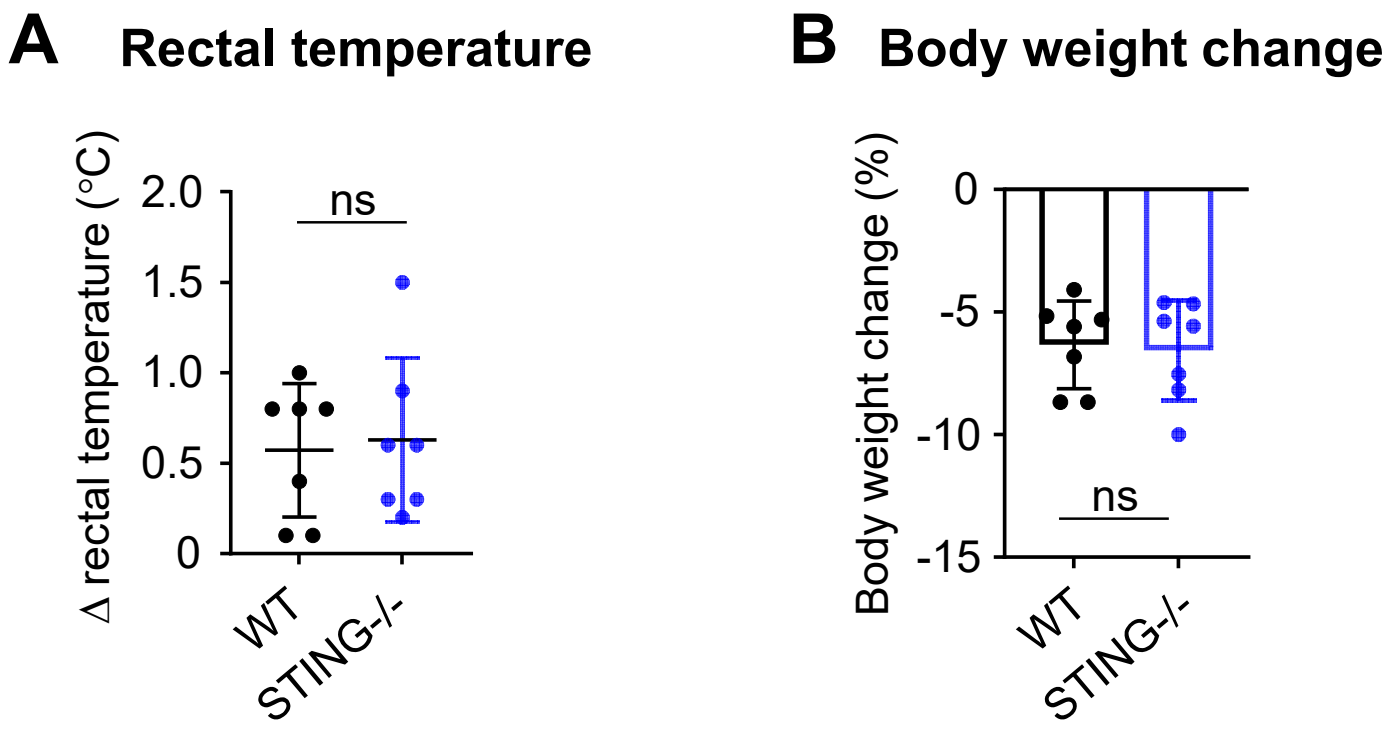

Figure S7. Effect of STING on induction of adverse reactions.
(A, B) STING knockout (STING-/-) or wild-type (WT) mice were vaccinated intramuscularly with 20 μg mRNA-LNP. After vaccination, (A) change in rectal body temperature at 6 h, and (B) body weight changes over 24 h were measured. (A, B) $n = 7$. Data are pooled results of two independent experiments. Data are expressed as means ± SD; ns, not significant by Student's *t*-test.

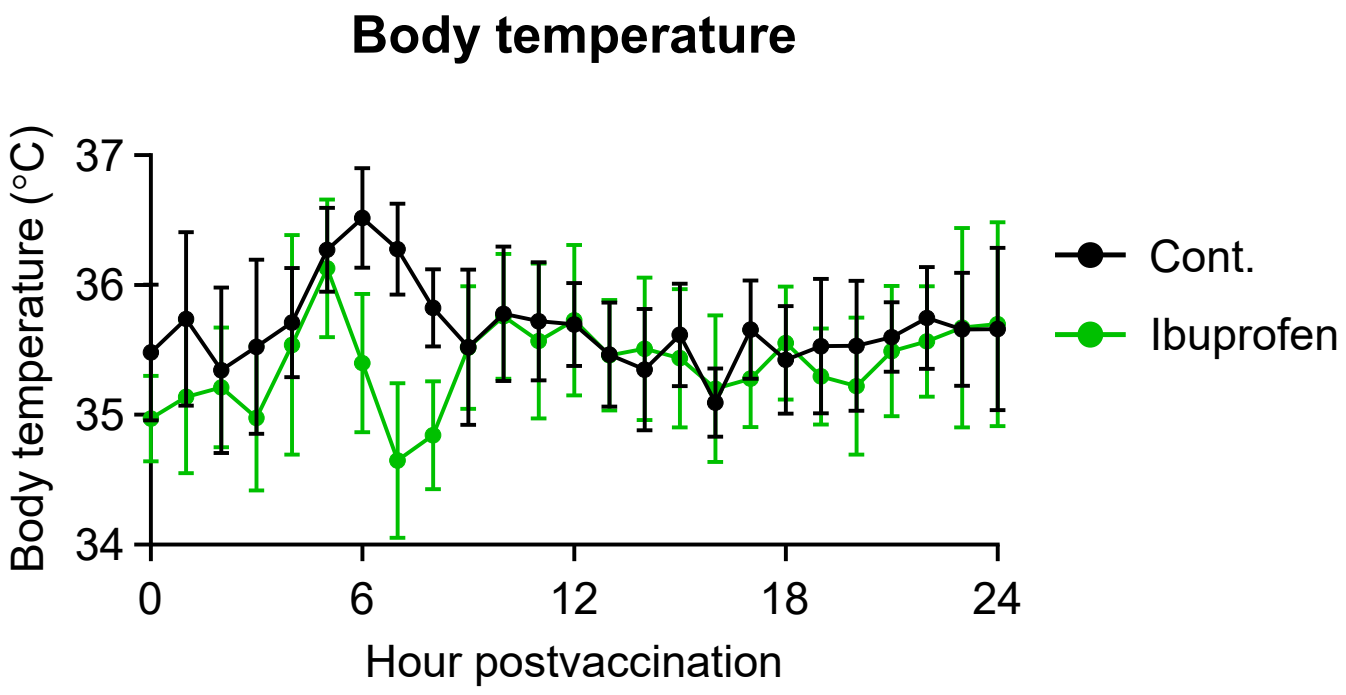

Figure S8. Effect of prostaglandin E2 on the induction of adverse reactions after the prime vaccination.
Mice were vaccinated intramuscularly with 20 μg mRNA-LNP, along with ibuprofen or 10% DMSO as a control, administered 30 min before and 6 h after vaccination. After vaccination, body temperature over 24 h was measured. n = 8. Data are pooled results of two independent experiments. Data are expressed as means ± SD.

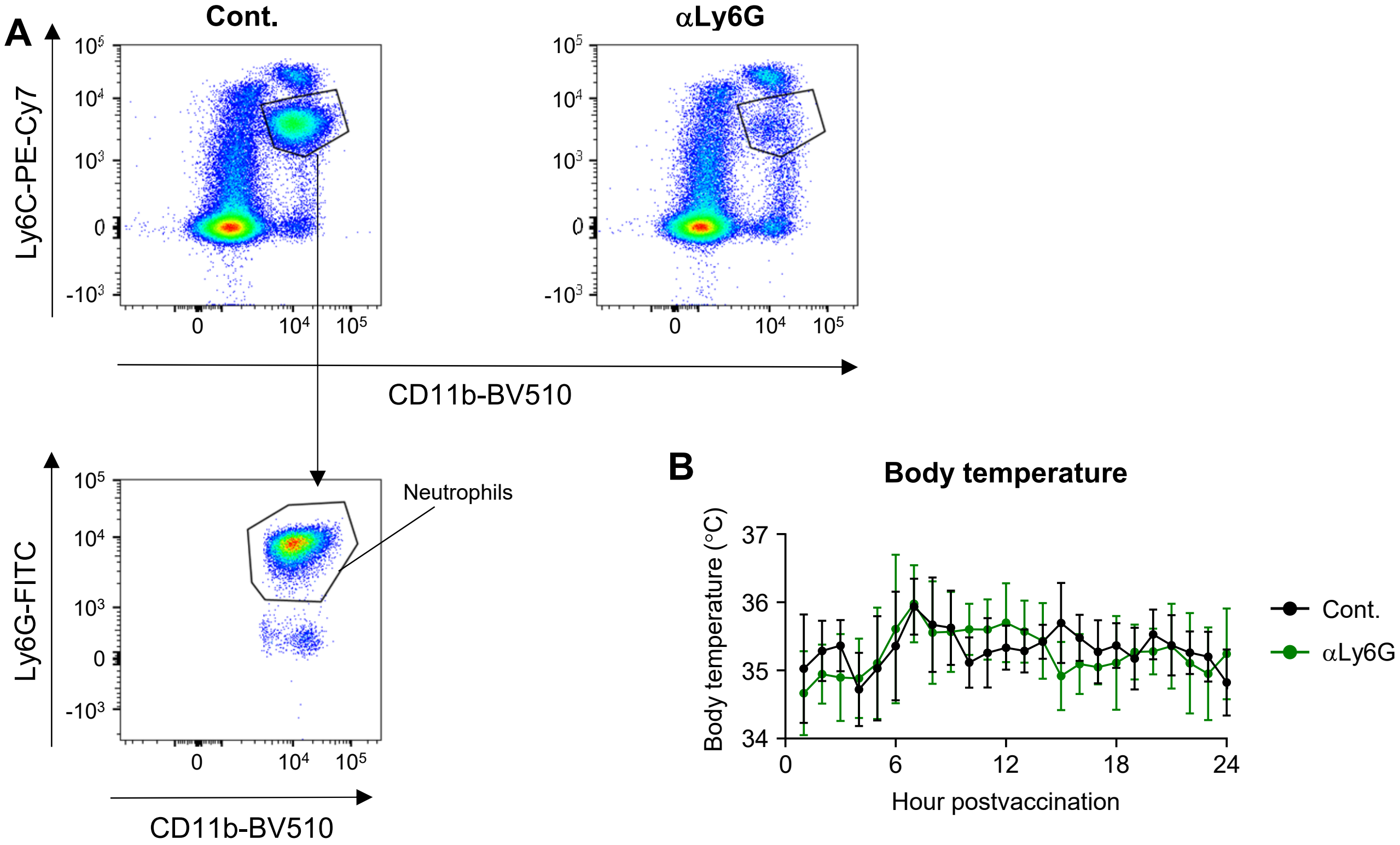

Figure S9. Effect of neutrophils on the induction of adverse reactions at the prime vaccination.
(A) Representative flow cytometry plots of CD11b$^+$ Ly6C$^+$ Ly6G$^+$ neutrophils gating on live CD45$^+$ cells in the blood are shown 1 day after the final administration of anti-Ly6G antibody. (B) Mice were administered anti-Ly6G antibody 2 days and 1 day before mRNA-LNP vaccination and were then vaccinated intramuscularly with 20 μg mRNA-LNP. After vaccination, body temperature over 24 h was measured. (B) n = 7. (B) Data are pooled results of two independent experiments. Data are expressed as means ± SD.

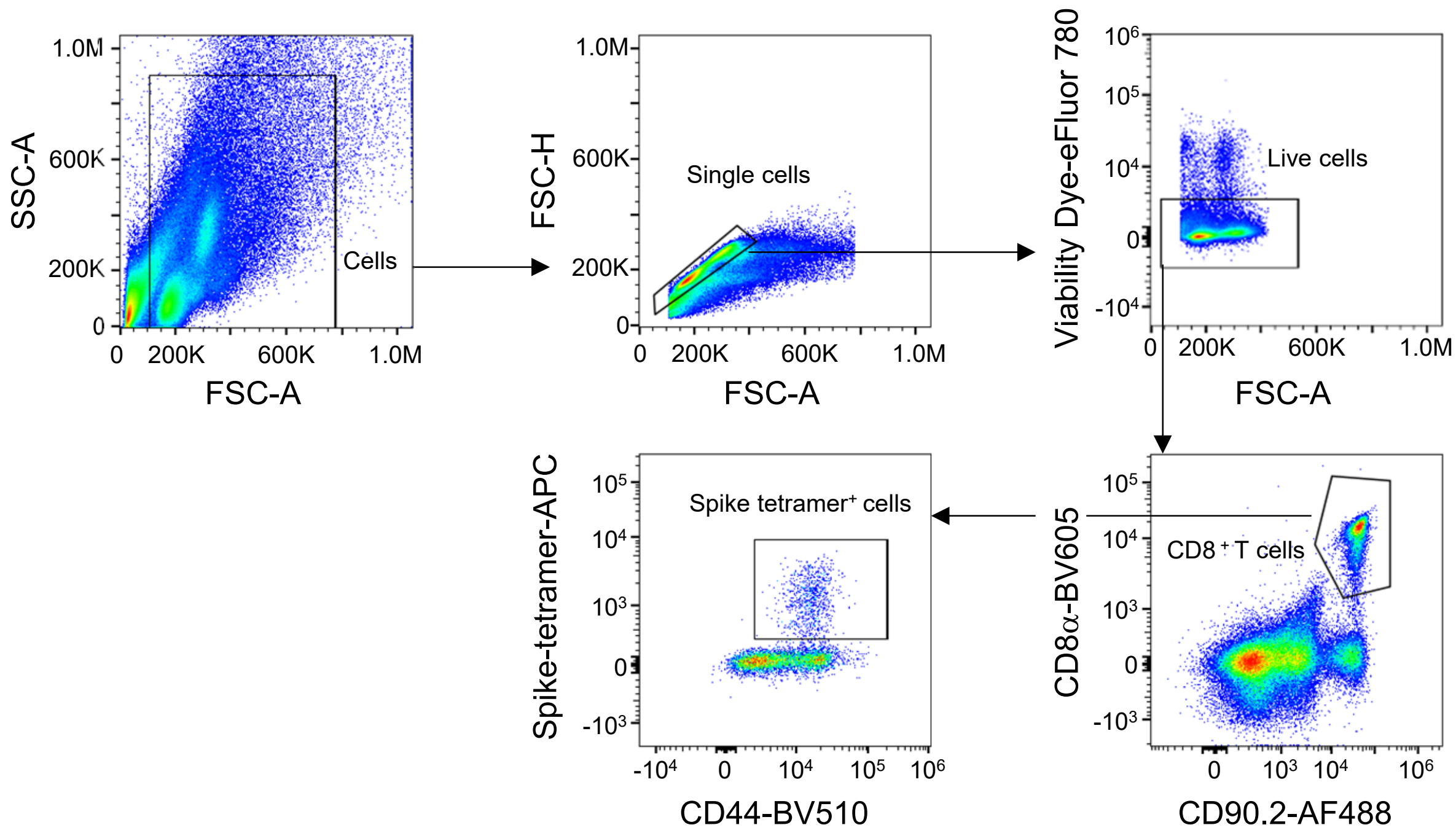

Figure S10. Gating strategy for spike-specific CD8⁺ T cells in blood.
Gating strategy for spike-specific CD8⁺ T cells in blood after 20 µg mRNA-LNP vaccination.

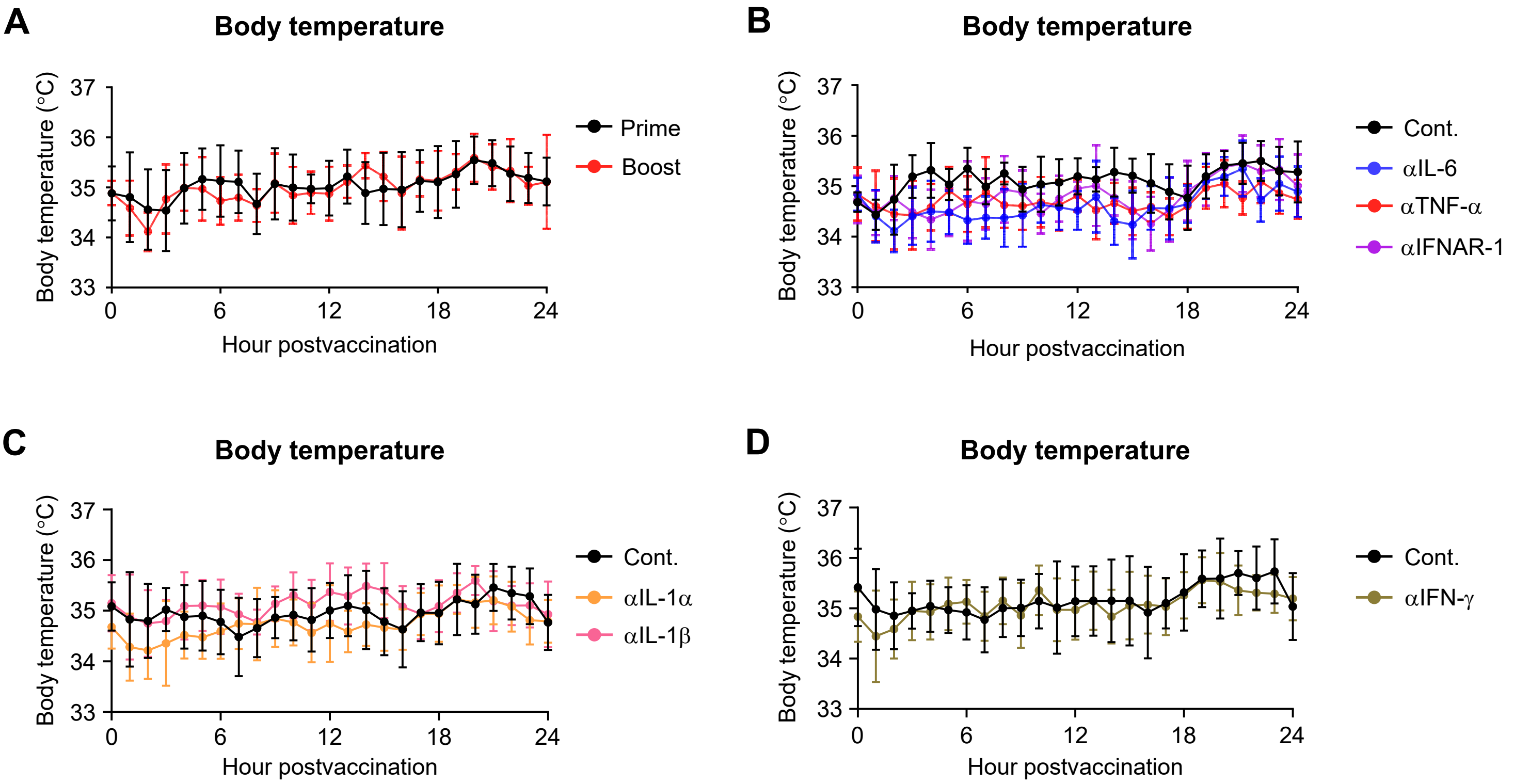

Figure S11. Effect of cytokines on fever after the boost vaccination.
Mice were vaccinated intramuscularly with 20 μg mRNA-LNP on day 0 and 21. (B-D) Mice were administered neutralizing antibody 24 h prior to the boost mRNA-LNP vaccination. (A-D) After vaccination, body temperature over 24 h was measured. (B) PBS was used as a control for neutralizing antibody. (C, D) Isotype control antibody or PBS was used as a control for neutralizing antibody. (A) n = 6–7, (B) n = 7–8, (C) n = 8–12, (D) n = 10–11. (A-D) Data are pooled results of two independent experiments. (C, D) Because of the experimental grouping, some data for the Control groups are shared. Data are expressed as means ± SD.

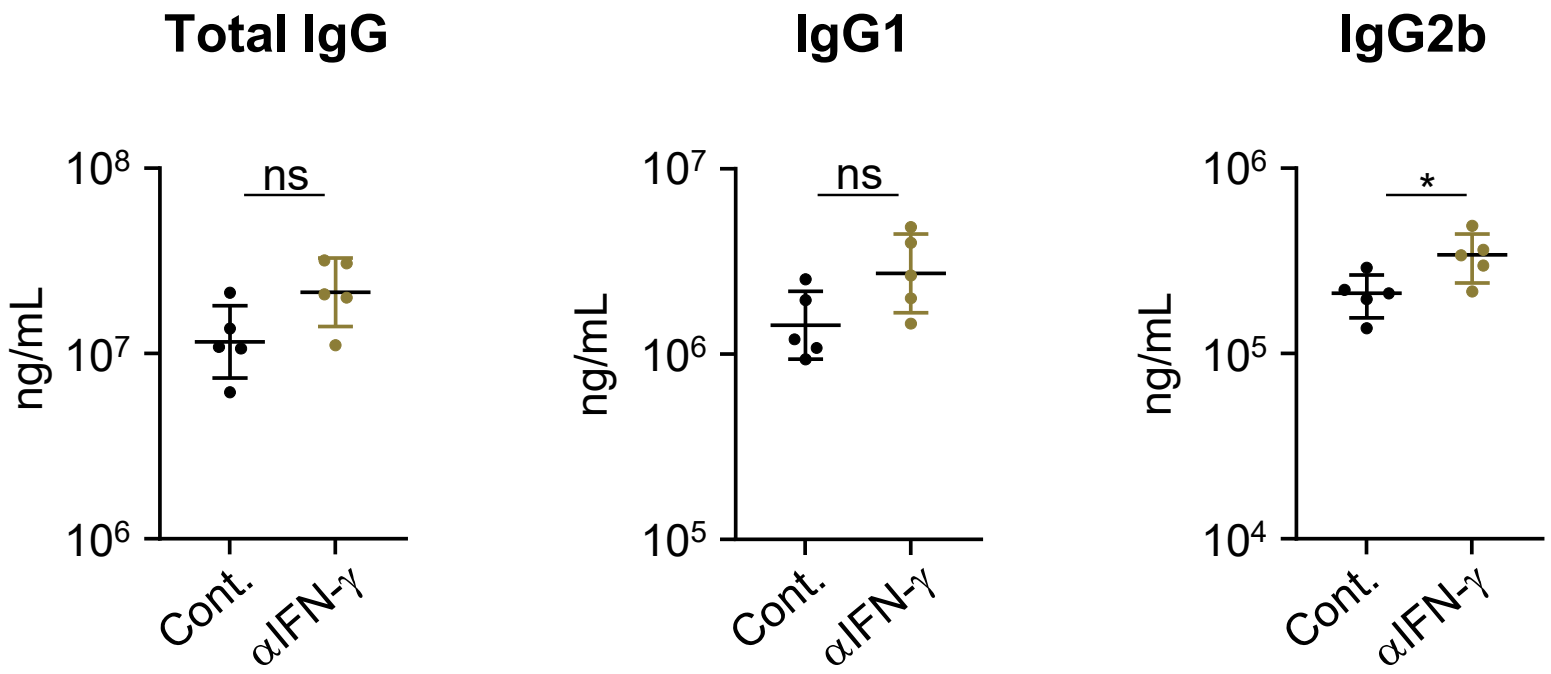

Figure S12. Effects of IFN-γ on antigen-specific antibody responses after the boost vaccination.
Mice were vaccinated intramuscularly with 20 μg mRNA-LNP on day 0 and day 21. They were administered neutralizing antibody 24 h before the boost vaccination. Concentrations of spike-specific IgG, IgG1, and IgG2b in plasma were assessed 9 days after the boost vaccination using ELISA. n = 5. Data are expressed as means ± SD; *P<0.05; by Student's t-test, ns, not significant.

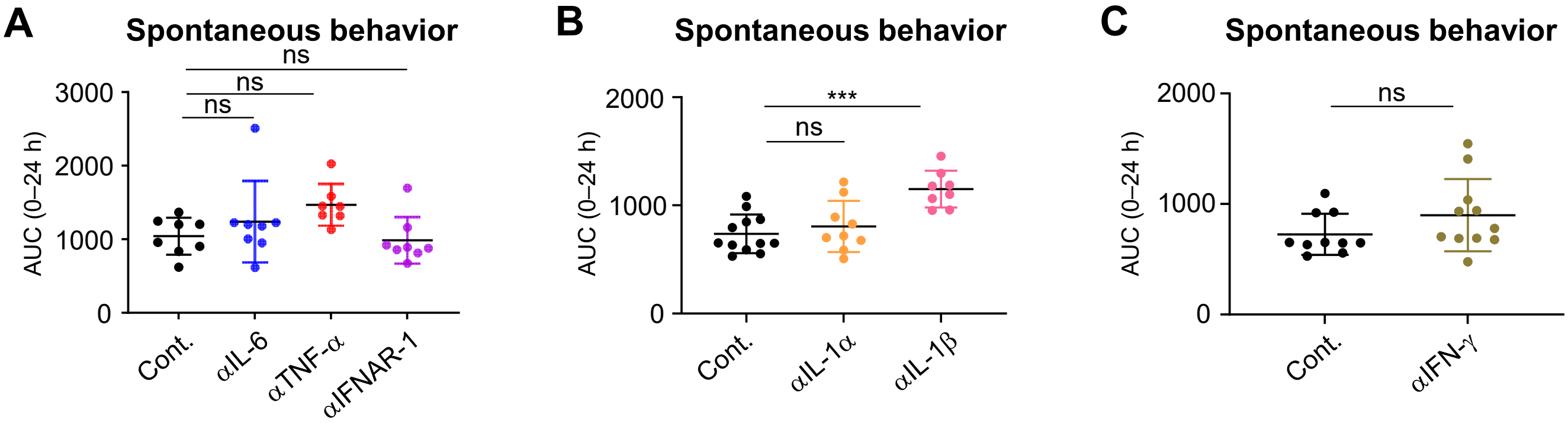

Figure S13. Effect of cytokines on spontaneous behavior after the boost vaccination.

Mice were vaccinated intramuscularly with 20 µg mRNA-LNP on day 0 and 21. Mice were administered neutralizing antibody 24 h prior to the boost mRNA-LNP vaccination. After vaccination, area under the curve (AUC) for behavior change over 24 h was measured. (A) PBS was used as a control for neutralizing antibody. (B, C) Isotype control antibody or PBS was used as a control for neutralizing antibody. (A) n = 7–8, (B) n = 8–12, (C) n = 10–11. Data are pooled results of two independent experiments. (B, C) Because of the experimental grouping, some data for the Control groups are shared. Data are expressed as means ± SD; ***P<0.001; (A, B) by Dunnett's multiple comparison test vs. Control, or (C) by Student's t-test. ns, not significant.

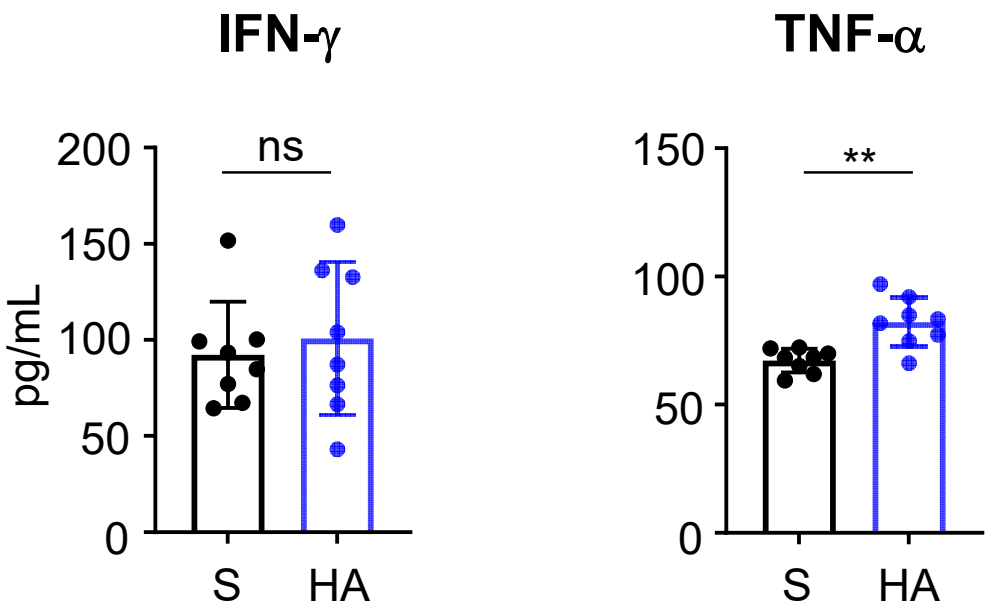

Figure S14. Blood cytokine levels after the prime vaccination.
Mice were vaccinated intramuscularly with 20 μg mRNA-LNP encoding spike or HA protein. IFN-γ and TNF-α levels in plasma were assessed 6 h after the prime vaccination. n = 8. Data are expressed as means ± SD; **P<0.01 by Student's t-test. ns, not significant.

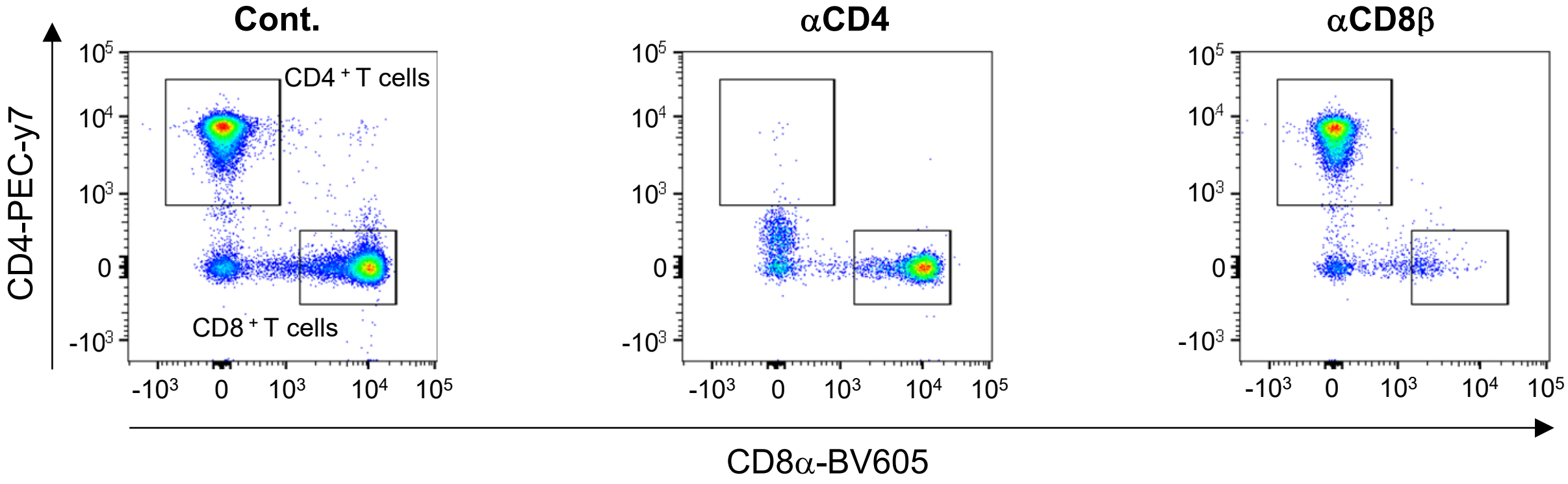

Figure S15. Flow cytometry plots of CD4⁺ or CD8⁺ T cells after the administration of anti-CD4 or anti-CD8 antibody. Representative flow cytometry plots of CD4⁺ or CD8⁺ T cells gating on live CD90.2⁺ cells in the blood 1 day after the administration of anti-CD4 or anti-CD8 antibody are shown.

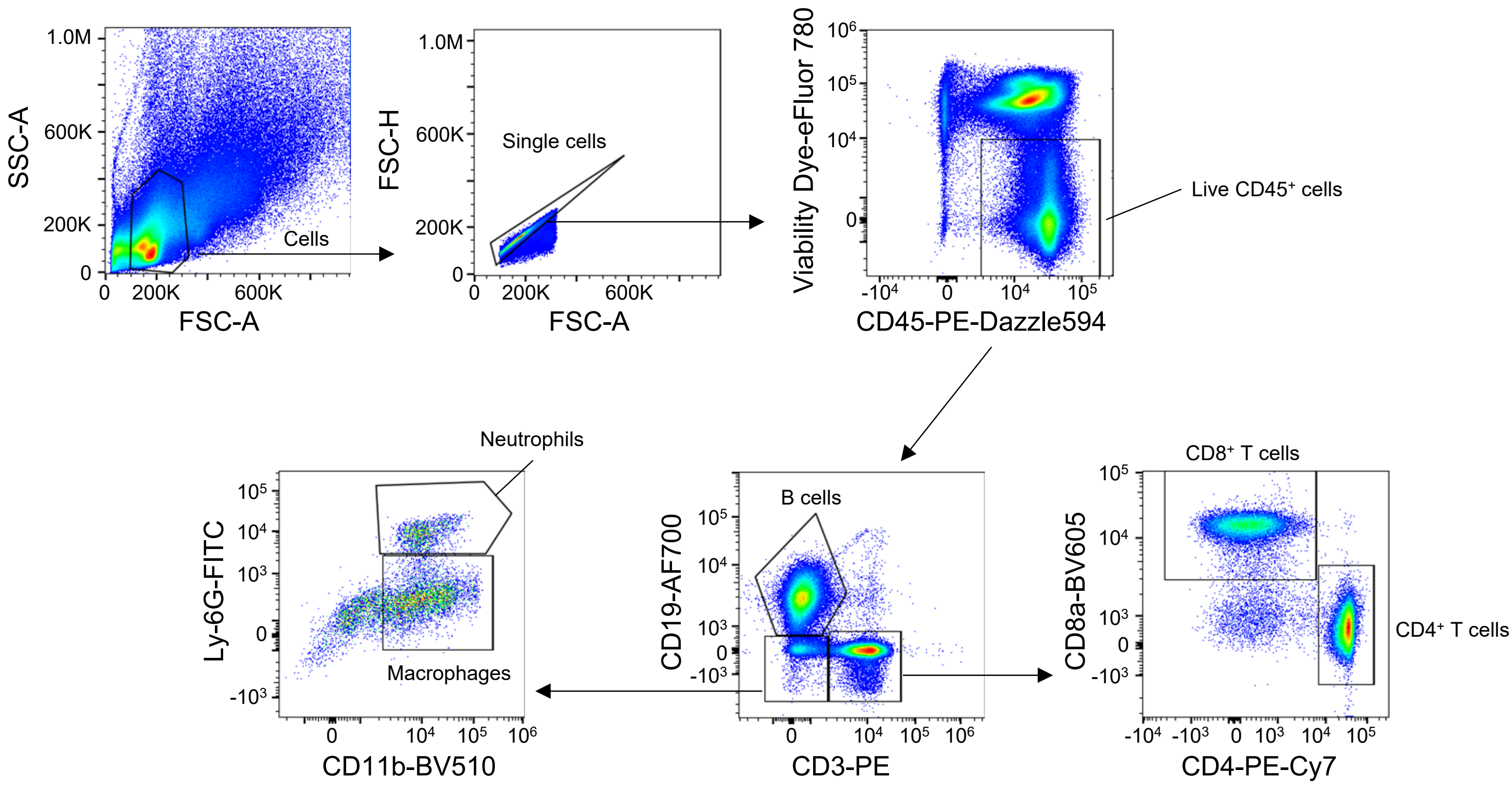

Figure S16. Flow cytometry analysis of TNF-α producing cells in spleen.
Gating strategy for TNF-α$^+$ cells in the spleen after restimulation with spike protein.

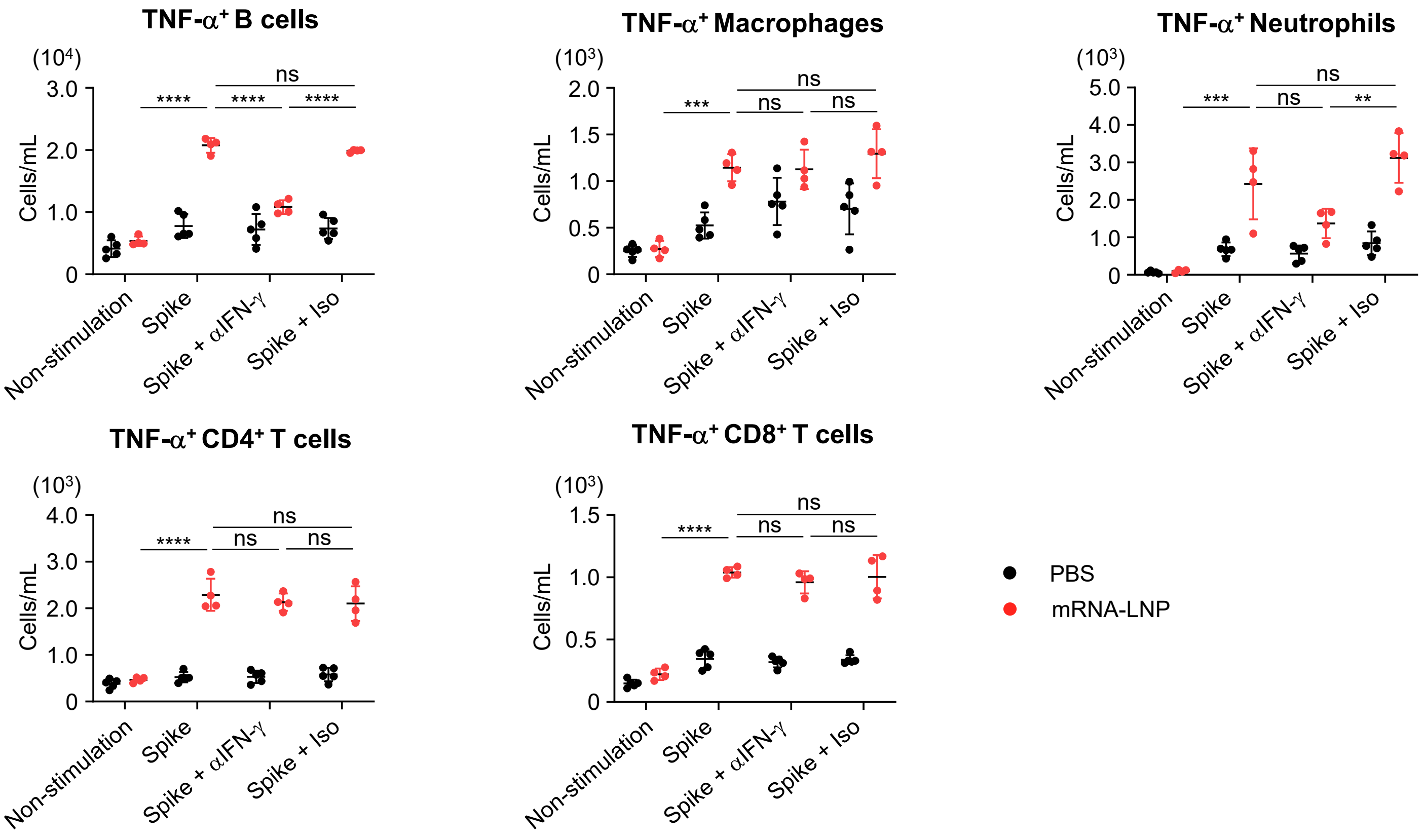

Figure S17. Effects of IFN-γ on TNF-α producing cells in spleen.

Mice were vaccinated intramuscularly with 20 μg mRNA-LNP, and after 21 days, splenocytes were stimulated with spike protein in the absence or presence of anti-IFN-γ antibody. Number of TNF-α+ B cells, macrophages, neutrophils, CD4+ T cells, and CD8+ T cells in the spleen was assessed after the stimulation using flow cytometry. *n* = 4–5. Data are expressed as means ± SD; $^{**}P<0.01$, $^{***}P<0.001$, $^{****}P<0.0001$; Tukey's multiple comparison test in mRNA-LNP group. ns, not significant.

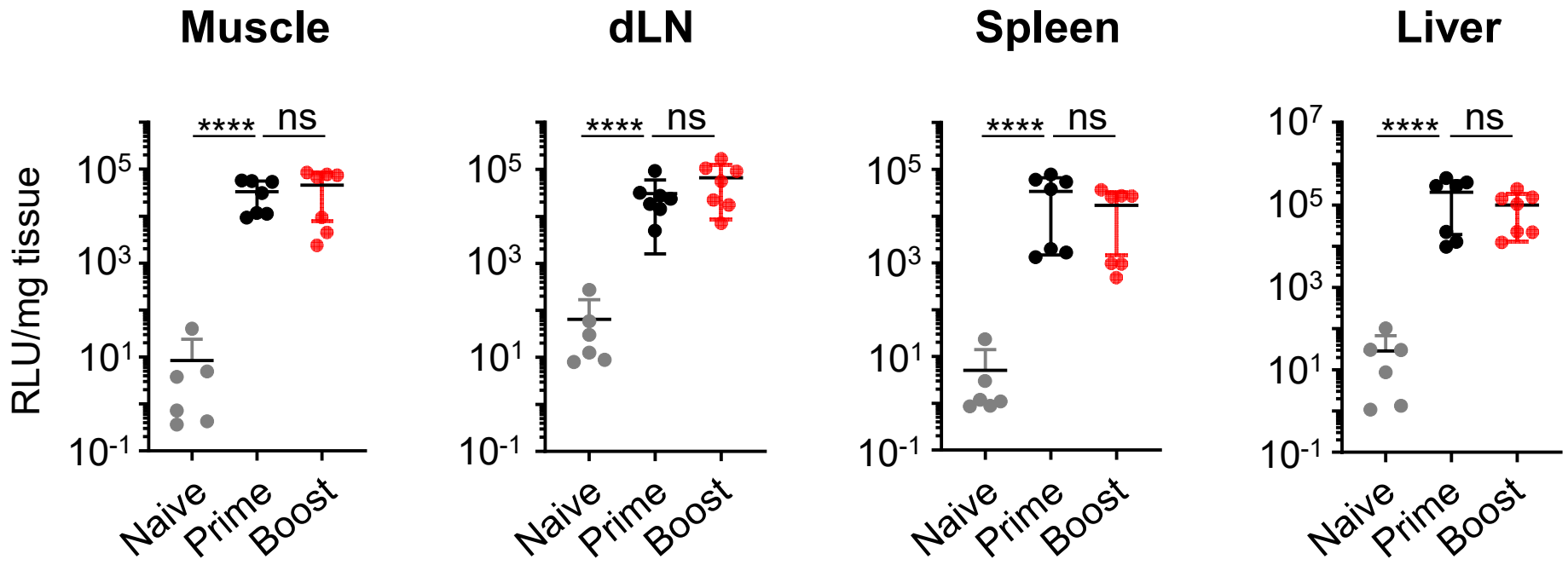

Figure S18. mRNA-LNP kinetics using mRNA encoding luciferase in various tissues.
Mice were vaccinated intramuscularly with 20 μg mRNA-LNP encoding luciferase on day 0 and 21. Relative light units (RLU) of luciferase were assessed 6 h after the prime and boost vaccination. n = 6–7. Data are pooled results of two independent experiments. Data are expressed as means ± SD; **** P<0.0001 by Dunnett's multiple comparison test vs. prime. ns, not significant.

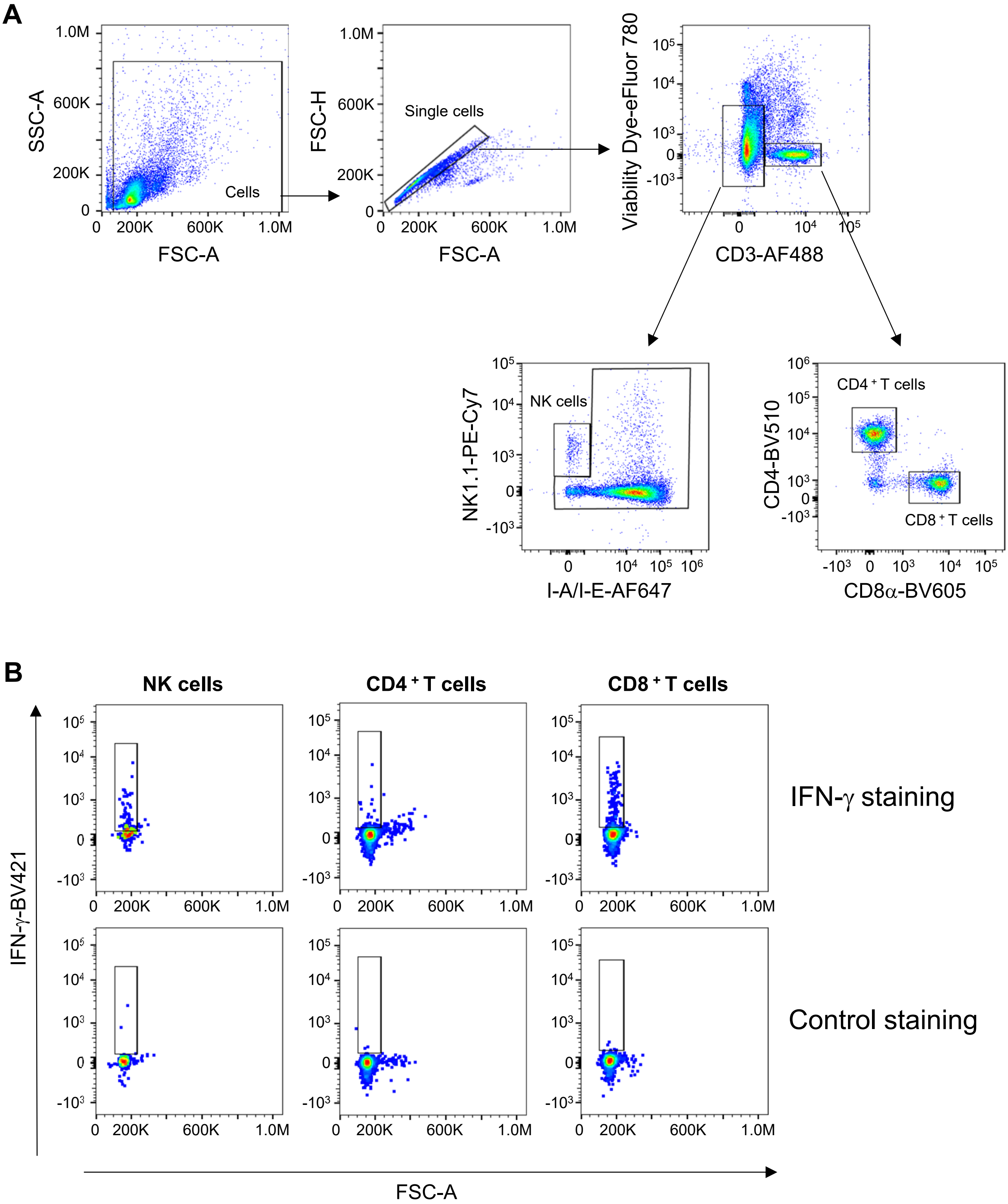

Figure S19. Flow cytometry analysis of IFN-γ–producing cells.
(A) Gating strategy for IFN-γ^+ cells in the spleen after mRNA-LNP vaccination. (B) Representative flow cytometry plots of IFN-γ^+ NK, IFN-γ^+ $CD4^+$ T, and IFN-γ^+ $CD8^+$ T cells in the spleen.

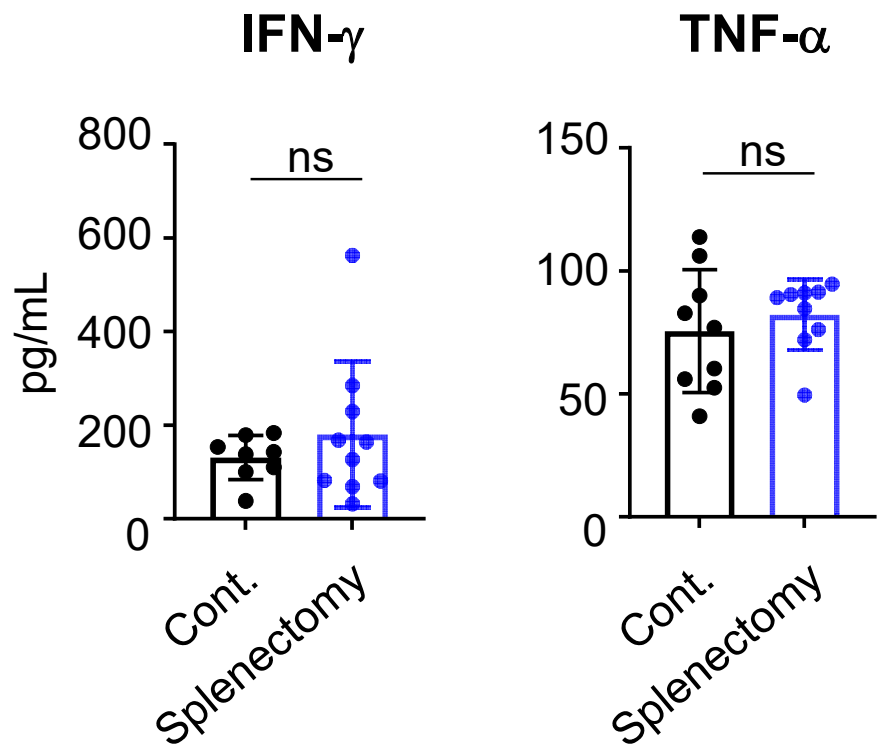

Figure S20. Effects of splenectomy on IFN-γ and TNF-α after the prime vaccination.
Mice were splenectomized 3 weeks before the prime vaccination and were vaccinated intramuscularly with 20 μg mRNA-LNP. IFN-γ and TNF-α levels in plasma were assessed 6 h after the prime vaccination. n = 9–10. Data are pooled results of two independent experiments. Data are expressed as means ± SD; ns, not significant by Student's t-test.

Table S1. Antibodies used for flow cytometry analysis.

Evaluating of inflammatory cells in muscle

**Antibody**	Clone	Source
Anti-mouse CD16/CD32 antibody	93	BioLegend
FITC anti-mouse Ly6G antibody	1A8	BioLegend
Alexa Fluor 700 anti-mouse I-A/I-E antibody	M5/2	BioLegend
Brilliant Violet 510 anti-CD11b antibody	M1/70	BioLegend
Brilliant Violet 421 anti-mouse CD45 antibody	30-F11	BioLegend
PE-Cy7 anti-mouse Ly6C antibody	HK1.4	BioLegend

Evaluating blood spike-specific $CD8^+$ T cells by flow cytometry

**Antibody**	Clone	Source
Alexa Fluor 488 anti-mouse CD90.2 antibody	53-2.1	BioLegend
Brilliant Violet 510 anti-CD44 antibody	IM7	BioLegend
Brilliant Violet 605 anti-mouse CD8α antibody	53-6.7	BioLegend

Evaluating IFN-γ–producing cells in spleen by flow cytometry

**Cell surface antigen staining**	Clone	Source
Anti-mouse CD16/CD32 antibody	93	BioLegend
Alexa Fluor 488 anti-mouse CD3ε Antibody	145-2C11	BioLegend
Alexa Fluor 647 anti-mouse I-A/I-E antibody	M5/2	BioLegend
Brilliant Violet 510 anti-mouse CD4 antibody	RM4-5	BioLegend
Brilliant Violet 605 anti-mouse CD8α antibody	53-6.7	BioLegend
PE-Cy7 anti-mouse NK1.1 antibody	PK136	BioLegend
**Intracellular cytokine staining**		
Brilliant Violet 421 anti-mouse IFN-γ antibody	XMG1.2	BioLegend

Effect of IFN-γ on TNF-α–producing cells

**Cell surface antigen staining**	Clone	Source
Anti-mouse CD16/CD32 antibody	93	BioLegend
FITC anti-mouse Ly6G antibody	1A8	BioLegend
Alexa Fluor 700 anti-mouse CD19 antibody	6D5	BioLegend
Brilliant Violet 510 anti-CD11b antibody	M1/70	BioLegend
Brilliant Violet 605 anti-mouse CD8α antibody	53-6.7	BioLegend
PE anti-mouse CD3 antibody	17A2	BioLegend
PE-dazzle594 anti-mouse CD45 antibody	30-F11	BioLegend
PE-Cy7 anti-mouse CD4 antibody	GK1.5	BioLegend
**Intracellular cytokine staining**		
Alexa Fluor 647 anti-mouse TNF-α antibody	MP6-XT22	BioLegend

Table S2. Primer sequences used for real time-PCR.

Gene	5′ primer	3′ primer
Il6	5′-CTGTAGCTCATTCTGCTCTGGA-3′	5′-CAACTGGATGGAAGTCTCTTGC-3′
Tnfa	5′-CCTCTCATGCACCACCATCAA-3′	5′-TTCTGAGACAGAGGCAACCTG-3′
Ifna	5′-GGACTTTGGATTCCCGCAGGAGAAG-3′	5′-GCTGCATCAGACAGCCTTGCAGGTC-3′
Ifnb	5′-AACCTCACCTACAGGGCGGACTTCA-3′	5′-TCCCACGTCAATCTTTCCTCTTGCTTT-3′
Ifng	5′-GGATGCATTCATGAGTATTGC-3′	5′-CCTTTTCCGCTTCCTGAGG-3′
Il1a	5′-CGTGTTGCTGAAGGAGTTGC-3′	5′-TCTGGATAAGCAGCTGATGTGA-3′
Il1b	5′-TCTTTGAAGTTGACGGACCC -3′	5′-TGAGTGATACTGCCTGCCTG-3′
Cox2	5′-TGAGCAACTATTCCAAACCAGC -3′	5′-GCACGTAGTCTTCGATCACTATC -3′
Gapdh	5′-CAGGTTGTCTCCTGCGACTT-3′	5′-AGCCGTATTCATTGTCATACCAGG-3′

Table S3. Antibodies used for neutralizing cytokines.

**Antibody**	Clone	Source
Anti-mouse IL-6-InVivo	MP5-20F3	Selleck
Anti-mouse TNF-α-InVivo	XT3.11	Selleck
Anti-mouse IFNAR-1-InVivo	MAR1-5A3	Selleck
Anti-mouse IFN-γ (XMG1.2)-InVivo	XMG1.2	Selleck
InVivoMAb anti-mouse IL-1α	ALF-161	Bio X Cell
InVivoMAb anti-mouse/rat IL-1β	B122	Selleck
Rat IgG1 isotype control-InVivo	HRPN	Selleck
Mouse IgG1 isotype control-InVivo	MOPC-21	Selleck/ Bio X Cell
InVivoMAb polyclonal Armenian hamster IgG		Bio X Cell

Table S4. Antibodies used for ELISA.

**Antibody**	Clone	Source
SARS-CoV-2 Spike RBD Antibody (IgG1)	1035753	R&D Systems
SARS-CoV-2 Spike RBD Antibody (IgG2b)	1035709	R&D Systems
Goat Anti-Mouse IgG (H+L)-HRP		Southern Biotech
Goat Anti-Mouse IgG1, Human ads-HRP		Southern Biotech
Goat Anti-Mouse IgG2b, Human/Bovine/Horse SP ads-HRP		Southern Biotech
